# Dynamics around small irregularly shaped objects modeled as a mass dipole

**DOI:** 10.1038/s41598-024-61821-w

**Published:** 2024-05-23

**Authors:** Ahmed A. Abozaid, M. Radwan, A. H. Ibrahim, A. Bakry

**Affiliations:** 1 Astronomy and Meteorology Department, Faculty of Science Al-Azhar University, Cairo, Egypt; 2 Astronomy and Space Science Department, Faculty of Science Cairo University, Cairo, Egypt

**Keywords:** Astronomy and astrophysics, Aerospace engineering

## Abstract

In this work, we investigate the dynamics of a spacecraft near two primary bodies. The massive body is considered to have a spherical shape, while the less massive one is elongated and modeled as a dipole. The dipole consists of two connected masses, one is spherical and the other is an oblate spheroid. The gravitational potential of the elongated body is determined by four independent parameters. To study the dynamics, we construct the equations of motion of a spacecraft with negligible mass under the effect of the current force model. The existence and locations of the equilibrium points are analyzed for various values of the system parameters. We found that the existence and locations of the points are affected by the system parameters. Also, we studied the linear stability of the equilibrium points. We found some stable collinear points when the oblateness parameter is negative, otherwise the points are not stable. We used the curves of zero velocity to identify the regions of allowed motion. Furthermore, we discussed the 2001 SN263 asteroid system and found some stable collinear points when the oblateness parameter is negative. In addition, the triangular points of the system are stable in a linear sense.

## Introduction

Great efforts are being made to discover small bodies within the solar system, such as asteroids and comets. The interest in studying these minor bodies has returned once again to the focus of international institutions after the successful launch of the Rosetta space mission. This scientific mission aimed to explore the materials, physical properties, and environments of these small celestial bodies. This efforts motivate and encourage the development of space explorations and expand our knowledge about the origin of our solar system^1^.

Currently, some space missions continue to work to study minor bodies like the Lucy mission launched in 2021, and others are planned to start in a few years. Therefore, understanding and studying the orbital dynamics around these bodies is essential. However, studying the dynamics around these bodies is complex due to their peculiar rotations and non-spherical shapes^2^ . Since these minor celestial bodies typically have irregular shapes, thus considering their attraction potentials as a small perturbation of the central gravitational field is often unsuitable. It is essential to construct a simple approximation for their gravitational fields while keeping the characteristics of motion in close proximity to these bodies^3^. There are several mathematical models have been constructed in order to represent the gravitational field of minor bodies with irregular shapes. Due to the shapes of these bodies, we can not apply the common spherical harmonic model of the gravitational field because of the slow convergence or even divergence of functions near the surface of these bodies^4^. Among the common mathematical models, the ellipsoidal harmonic model suggested by Hobson^5^ and then modified by Pick et al.^6^, the spherical harmonic one was adopted to describe the gravitational field of the asteroid Vesta^7^. Some alternative methods are also presented to investigate the dynamics around the elongated asteroids or comets. Among them, the rotating mass dipole^8^, the massive straight segment^9^ , the double segment model^10^ , or the simple dumbbell-shaped body model^11^. Furthermore, Zeng et al.^12^ proposed a simplified dipole segment model. This model consists of a massive straight segment and two point masses at the extremities of the segment. Using the simple potential function associated with the proposed model, they identified five topological cases with different sets of system parameters. In addition, the authors investigated the positions, stabilities, and variation trends of the system equilibrium points in a parametric way.

Lagrange equilibrium points are positions in space in which the spacecraft has zero acceleration and zero velocity. These points are located in regions where the gravitational perturbations are minimal, so we can reduce the fuel needed for maneuvers and station-keeping^13^. The equilibrium points of irregularly shaped minor bodies play an important role in investigating the dynamic behaviors of spacecraft around these bodies^14^.

Different techniques have been used to address dynamic systems. Usually, quantitative methods, either numerical or analytical, give deep insight into the dynamic behavior of the systems. However, in most cases, dynamical systems described by differential equations are very complex. Thus, an analytical solution to the differential equations is not tractable. Also, numerical solutions are not valid for a very long interval of time. Here, to address the current problem more efficiently, we combine an analytical perturbed solution with a qualitative method. This method studies the geometric structure of the phase space portrait and deals with questions of stability etc.

Many researchers have been interested in studying the orbital dynamics of spacecraft that move around irregular minor celestial bodies. Mondelo et al.^15^ presented the four equilibrium solutions around the asteroid 4 Vesta asteroid and discussed their stability. Wang et al.^16^ used the polyhedral method to investigate the location and stability of the equilibrium points of 23 minor objects. They found four equilibrium points outside the elongated bodies. Yong et al.^17^ presented a simplified dynamical model for non-axisymmetric elongated asteroids. This model consists of three particles and two massless rigid rods. The authors applied the model to some realistic asteroids. They demonstrated that the topological cases of the Lagrange equilibrium points are not changed by the use of the proposed model.

Recently, Santos et al.^18^ investigated the qualitative orbital dynamics in close proximity to an asteroid with an arched shape using a tripole model. The authors applied their results to some real systems, such as 33 Eros. They found that, due to the arched shape of the asteroid, the curves around the rotating mass tripole have significant changes. Liu et al.^19^ studied the orbital dynamics with the gravitational potential of 93 Minerva utilizing an irregularly shaped model. The authors found five equilibrium points around the asteroid 93 Minerva, one of which is internal and four are external. Also, they studied the changes in position, number, and topological case of the Lagrangian points when changing the density and the spin speed. Furthermore, they demonstrated the existence of stable orbits around the asteroid 93 Minerva. Zeng and Liu^20^ proposed a new method to obtain natural periodic orbits near irregularly shaped asteroids. The method is based on the optimal control framework with respect to a general form of the irregular gravitational field. The authors identified three types of periodic orbits. These orbits are the Lyapunov orbit around the collinear point, the equatorial retrograde orbit, and the inclined orbit. Also, Zhang et al.^21^ introduced the dipole segment model and its equilibrium points. The authors examined numerically the stability of the two triangular equilibrium points of the system. They illustrated new types of periodic orbits, including their orbital shapes, periods, and the Jacobi integral.

Most recently, Li et al.^22^ investigated the geophysical and orbital environments of the asteroid 2016 HO3 to facilitate a potential mission design. They examined the geometric and geopotential topographies of 2016 HO3 using different shape models. Then, the authors studied the periodic orbits around 2016 HO3 in the asteroid-fixed frame and the Sun-asteroid frame taking into account the solar radiation pressure. This work can serve as a reference for the exploration of other small-sized fast-rotating objects similar to 2016 HO3. Furthermore, Vincent et al.^23^ studied numerically a version of the synchronous restricted three-body problem. The authors considered the massive primary as an oblate spheroid, while the secondary one is an elongated asteroid. They investigated the existence, and linear stability of the libration points for different combinations of the system parameters. They observed that the perturbing forces have significant effects on the positions and stability of the libration points as well as the allowed regions of motion.

Critical scientific questions about small objects, such as structure, and formation can only be addressed by satellite missions that approach these objects^24^. Thus, understanding the behavior of the orbital dynamics of artificial satellites close to these objects is crucial to space missions. Also, using new realistic dynamical models contributes to obtaining highly accurate results. The above facts are the motivations of the current work. The focus of the current work is the study of the dynamics around small elongated celestial bodies. We used a new model to approximate irregularly shaped bodies. The paper extends the work performed by Idrisi et al.^25^. In their research, they assumed that the rotating dipole mass is formed by two equal point masses. However, in the present work, we assumed the rotating dipole to have a complex shape to obtain highly accurate results. Furthermore, we generalized the problem and investigated the existence and stability taking into account the parameters of the dipole model. The present work’s derived results can be a theoretical reference for future missions. The contents of the current paper are organized as follows: The next section introduces the dynamical equations of the system. After that, the existence and locations of the equilibrium points and their linear stability are analyzed for various values of the system parameters. Then, the curves of zero velocity are illustrated to show the topological structure around the two primaries along with the libration points. Finally, we studied the 2001 SN263 asteroid system by computing the locations of the equilibria and their stability besides the allowed regions of motion.

## Dynamical equations of motion

**Figure 1:**
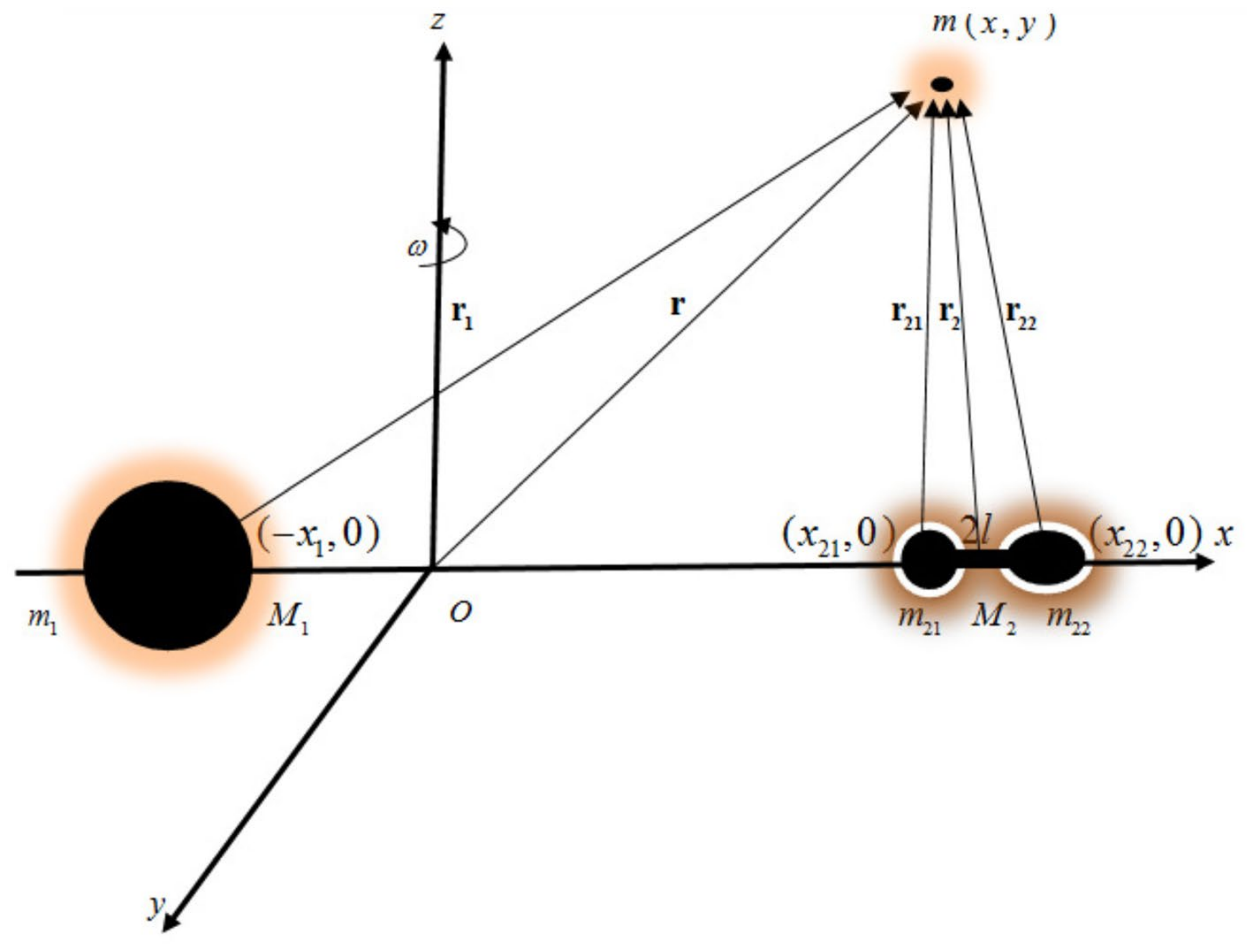
The geometry of the problem.

In the current work, we assume that the motion of the infinitesimal body is governed by the gravitational field of two primary bodies of masses $M_1$ and $M_2$. The two massive primaries rotate with an angular velocity $\mathbf \omega$ about their common center of mass. The infinitesimal body does not influence the dynamics of the two massive primaries. $M_1$ is assumed spherical, while $M_2$ is of irregular shape, $M_1> M_2$. The primary $M_2$ is modeled as a mass dipole composed of two masses $m_{21}$ and $m_{22}$, with total mass $M_2=m_{21}+m_{22}$. The mass $m_{21}$ is spherical, while $m_{22}$ is an oblate spheroid. The two masses are connected with a massless rod (Fig. 1).

To describe the dynamical behavior of the infinitesimal body, we use a body-fixed synodic frame *oxyz*. The origin of this reference frame is located at the barycenter of the system. ${{\textbf {r}}}_1$, ${{\textbf {r}}}_{21}$, ${{\textbf {r}}}_{22}$, and ${{\textbf {r}}}$ represent the position vectors of the infinitesimal body from the masses $m_1$, $m_{21}$, $m_{22}$, and the barycenter, respectively. The equatorial plane of the dipole coincides with the plane *oxy*, whereas the axis *ox* is collinear with the two primary bodies. The coordinates of the negligible mass with respect to the rotating coordinate system are given by (*x*, *y*), while the coordinates of the two massive primaries are $(-x_1,0)$, $(x_{21},0)$ and $(x_{22},0)$, respectively. Because the mass of the more massive primary is greater than that of the less massive one, thus we can define the mass ratio of the system as $\mu ^ \text {*}=\frac{m_{21}}{m_1+m_{21}+m_{22}}=\frac{\mu }{2}$. $\mu$ represents the known mass ratio of the classical restricted three-body problem. Therefore, the coordinates of the massive primaries are given by $x_1=2\mu ^*$, $x_{21}=1-2\mu ^*-l$ and $x_{22}=1-2\mu ^*+l$. 2*l* represents the distance between the two masses $m_{21}$ and $m_{22}$. Here, to facilitate the calculations and discussions we use dimensionless canonical units to describe the problem. The unit of mass is chosen such that the sum of the masses is equal to unity, $m_1+m_{21}+m_{22}=1$. Also, we define the unit of time such that the period of rotation of the mass dipole is equal to $2 \pi$. Furthermore, we choose the distance between $M_1$ and the center of mass of $M_2$ as the unit of distance.

Now, in the adopted rotating coordinate dimensionless system, the differential equations of motion of the small negligible mass *m*, in the (*x*, *y*) plane, under the effect of the gravitational potential of the primary bodies $M_1$ and $M_2$ are given by

1 $$\begin{aligned} \begin{array}{rl} {\dot{x}} - 2\omega {\dot{y}} = {\Omega _x}, \\ {\ddot{y}} + 2\omega {\dot{x}} = {\Omega _y} \end{array} \end{aligned}$$

where

2 $$\begin{aligned} \begin{array}{rl} \Omega =\frac{1}{2}{\omega ^2}\left( {{x^2} + {y^2}} \right) + k{\omega ^2}\left( {\frac{{\left( {1 - 2\mu ^* } \right) }}{{{r_1}}} + \frac{\mu ^* }{{{r_{21}}}} + \frac{\mu ^* }{{{r_{22}}}}\left( {1 + \frac{A_{}}{{2r_{22}^2}}\left( {1 - \frac{{3{z^2}}}{{r_{22}^2}}} \right) } \right) } \right) \end{array} \end{aligned}$$

where $\Omega _x$ and $\Omega _y$ represent the partial derivatives of the potential $\Omega$ with respect to *x* and *y*, respectively. The force ratio parameter $k= \frac{{GM}}{{\omega ^2}{d^3}}$ plays an important role in the dynamics of the problem since it represents the ratio between the gravitational and the centrifugal forces, with *d* the distance between the two primaries. When the ratio $k = 1$, the problem is identical to the classical circular restricted problem. If $k<1$, the centrifugal force is larger than the gravitational one and the primary massive bodies tend to move away from each other. Vice versa, in the case $k>1$, the primary bodies tend to approach each other^26^. $r_1$, $r_{12}$ and $r_{22}$ are the distances from the small mass *m* to the masses $m_1$, $m_{12}$ and $m_{22}$, respectively, and can be expressed as

3 $$\begin{aligned} \begin{array}{l} r_1^2 = {\left( {x + 2\mu ^* } \right) ^2} + {y^2}\\ \\ r_{21}^2 = {\left( {x + 2\mu ^* - 1 + l} \right) ^2} + {y^2}\\ \\ r_{22}^2 = {\left( {x + 2\mu ^* - 1 - l} \right) ^2} + {y^2} \end{array} \end{aligned}$$

where the perturbed angular velocity presented in Eq. (2) is no longer equal to unity and is given by

4 $$\begin{aligned} \omega = \sqrt{1 + \frac{3}{2}A_{}} \end{aligned}$$

where *A* represents the oblateness coefficients of $m_{22}$^27^

5 $$\begin{aligned} {A_{}} = \frac{{({{(P_{22}^e)}^2} - {{(P_{22}^p)}^2})}}{{5{d^2}}} \end{aligned}$$

where the parameter *P* is the spheroid primary radius. The superscript *e* represents the equatorial radius, and *p* is the polar radius.

The gradients of the effective potential in the plane (*x*, *y*), can be written as

6 $$\begin{aligned} \begin{array}{l} {\Omega _x} = {\omega ^2}\left( {x - k\left( \begin{array}{l} \frac{{\left( {1 - 2\mu ^* } \right) \left( {2\mu ^* + x} \right) }}{{r_1^3}} + \frac{{\mu ^* \left( { 2\mu ^* + x - 1 + l } \right) }}{{r_{21}^3}} + \frac{{\mu ^* \left( { 2\mu ^* + x- 1 - l } \right) }}{{r_{22}^3}}\left( {1 + \frac{{3A}}{{2r_{22}^2}} - \frac{{15A {z^2}}}{{2r_{22}^4}}} \right) \end{array} \right) } \right) \vspace{0.5cm} \\ {\Omega _y} = {\omega ^2}y\left( {1 - k\left( {\frac{{\left( {1 - 2\mu ^* } \right) }}{{r_1^3}} + \frac{\mu ^* }{{r_{21}^3}} + \frac{\mu ^* }{{r_{22}^3}}\left( {1 + \frac{{3A}}{{2r_{22}^2}} - \frac{{15A{z^2}}}{{2r_{22}^4}}} \right) } \right) } \right) \end{array} \end{aligned}$$

by using Eqs. (1), (2), then the Jacobian integral is

7 $$\begin{aligned} {{\dot{x}}^2} + {{\dot{y}}^2} = 2\Omega \left( {x,y} \right) - C \end{aligned}$$

where the integration constant *C* is an integral of the equation of motion and is called Jacobi’s constant.

## Equilibrium points of the dipole system

The libration points are locations of gravitational balance between the massive primary bodies. In other words, the equilibria of the dipole system can be found, in the rotating frame of reference, with zero values of both velocity and acceleration components in Eq. (8). In the classical restricted three-body problem, there exist five equilibrium points, out of which three collinear points are located on the x-axis, while the other two are located in the $x y-$plane and are known as triangular points. In the current section, we study the existence of equilibrium points in the equatorial plane of the dipole system in terms of their positions.

**Figure 2:**
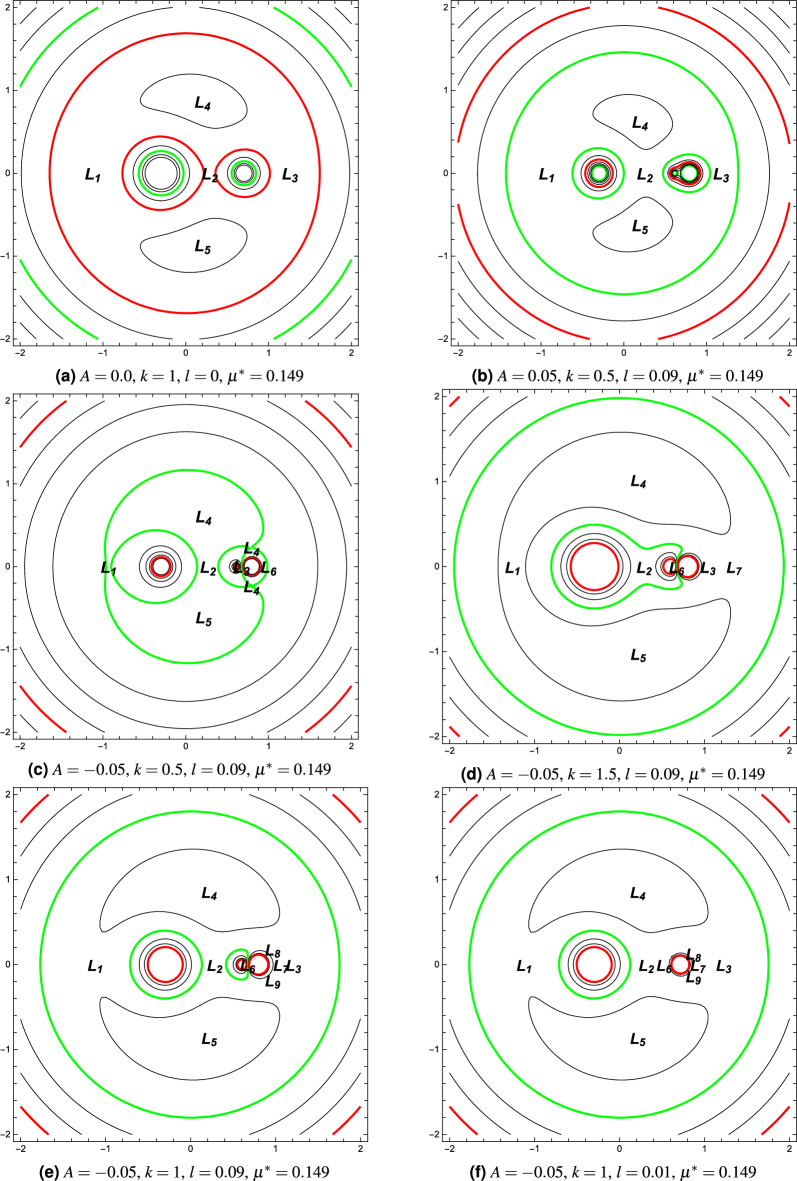
Equilibrium points and the zero-velocity curves around the dipole model with different parameters of (*k*, *A*, *l*).

The equations of motion of the system, Eq. (1), can be written as

8 $$\begin{aligned} \begin{array}{l} {\dot{x}} - 2\omega {\dot{y}} = {\omega ^2}x - {\omega ^2}k\left( \begin{array}{l} \frac{{\left( {1 - 2\mu ^* } \right) \left( {2\mu ^* + x} \right) }}{{r_1^3}} + \frac{{\mu ^* \left( { 2\mu ^* + x- 1 + l } \right) }}{{r_{21}^3}} + \frac{{\mu ^* \left( { 2\mu ^* + x- 1 - l } \right) }}{{r_{22}^3}}\left( {1 + \frac{{3A}}{{2r_{22}^2}}} \right) \end{array}\right) ,\vspace{0.3cm} \\ {\ddot{y}} + 2\omega {\dot{x}} = {\omega ^2}y\left( {1 - k\left( {\frac{{\left( {1 - 2\mu ^* } \right) }}{{r_1^3}} + \frac{\mu ^* }{{r_{21}^3}} + \frac{\mu ^* }{{r_{22}^3}}\left( {1 + \frac{{3A}}{{2r_{22}^2}}} \right) } \right) } \right) \end{array} \end{aligned}$$

The libration equilibrium points of the system can be obtained in the equatorial plane by equating the velocity and acceleration components to zero, $\Omega _x=0$ and $\Omega _y=0$, i.e. the right-hand side of Eq. (8), is set to zero

9 $$\begin{aligned} \begin{array}{l} \Omega _x = 0 \Rightarrow x - k\left( \begin{array}{l} \frac{{\left( {1 - 2\mu ^* } \right) \left( {2\mu ^* + x} \right) }}{{r_1^3}} + \frac{{\mu ^* \left( { 2\mu ^* + x - 1 + l } \right) }}{{r_{21}^3}} + \frac{{\mu ^* \left( { 2\mu ^* + x - 1 - l } \right) }}{{r_{22}^3}}\left( {1 + \frac{{3A}}{{2r_{22}^2}}} \right) \end{array} \right) \vspace{0.3cm} =0, \\ \Omega _y = 0 \Rightarrow y\left( {1 - k\left( {\frac{{\left( {1 - 2\mu ^* } \right) }}{{r_1^3}} + \frac{\mu ^* }{{r_{21}^3}} + \frac{\mu ^* }{{r_{22}^3}}\left( {1 + \frac{{3A}}{{2r_{22}^2}}} \right) } \right) } \right) =0. \end{array} \end{aligned}$$

Solving Eq. (9), we obtain two different types of solutions for the positions of the libration points. In the first case, when $x\ne 0$ and $y=0$, the solutions of this equation are called collinear solutions. In the second case, when $x\ne 0$ and $y\ne 0$, the solutions are non-collinear.

Here, we investigate, for the current system, the distribution of equilibrium points and the structure of the zero-velocity surfaces using numerical simulations. The curves of zero velocity determine the boundary regions in which the motion of the small body is permitted. We investigate the distribution of the points in the presence of the perturbing parameters, the oblateness coefficient *A*, the force ratio parameter *k*, and the distance *l*. To better study the problem and to show the significance of these parameters, different values of them are taken into consideration. In the solar system, the majority of planets are oblate spheroids and rotate in relatively stable states about their minor axes. As a consequence, the value of the oblateness coefficient *A* is positive for the primary bodies. However, in some cases without spinning, the fixed massive primaries can be prolate spheroids corresponding to a stable system. Therefore, the value of the oblateness coefficient can take values less than zero^28,29^. In the subsequent simulations, we used the values $A \in \{ -0.05, 0, 0.05\}$.

Figure 2a, depicts the simplified case corresponding to $A = 0$, $k = 1$, $l = 0$, and $\mu ^* = 0.149$. It is observed that the figure is similar to that of the restricted three-body problem. Figure 2b,c, illustrate the distribution of equilibrium points along with the zero-velocity curves with different *A*. In Fig. 2b, the oblateness coefficient of the dipole is $A = 0.05$, $k = 0.5, l = 0.09$ and $\mu ^{*}=0.149$. We can notice from the figure that, five equilibrium points are obtained, $L_i (i = 1, 2, \ldots , 5)$. Three collinear points $(i = 1, 2, 3)$ and two triangular points $(i = 4, 5)$. The distribution of the libration points is slightly different from Fig. 2a. As clear from Fig. 2b, the transfer of the small body between some of the points is possible. Furthermore, the curves of zero velocity distinguish the regions where the motion of the small particle is permissible from the regions where the motion is not permitted. For instance, the motion around the two triangular points is not permissible. In Fig. (2c), when $A = -0.05$, we can see that the distribution of the equilibrium points and the structure of the curves of zero velocity are completely different. We notice from the figure that new distinct points appear. Also, the transfer between some of the points is still possible.

Figure 2d,e depict the effect of varying the force ratio parameter *k* on the distribution of equilibrium points and the shape of the contours. Two values for *k* are considered $(k = 1.5; k = 1 )$ and the rest of the parameters are $A = -0.05, \mu ^* = 0.149$, and $l = 0.09$. In the case $k=1.5$, we observe from Fig. 2d that several distinct equilibrium points exist. Also, we notice from the contours that the transfer of the small body between some of the points is allowed. In the second case when $k=1$, several new points appear near to the less massive primary. The number of points is sensitive to the values of the force ratio. Furthermore, we can observe a slight change in the curves of zero velocity between the two cases.

In Fig. 2e,f, we illustrate the effect of the distance parameter *l* on the distribution of the libration points and the surfaces of zero velocity. We consider two values for this parameter $(l = 0.09; l = 0.01)$, while $A = -0.05$, $k = 1$, and $\mu ^* = 0.149$. In both cases, we notice the appearance of several equilibrium points and the contour shapes are slightly different. The motion of the small body is forbidden around the points $L_4$ and $L_5$ and the points appear close to the dipole.

## The location of non-collinear equilibria

It is known that the velocity and acceleration components of the infinitesimal body are equal to zero at the equilibrium points^30^. These non-collinear equilibria are the solution of the equations $\Omega _x=0$, and $\Omega _y=0$, when $(x,y) \ne (0, 0)$, then

10 $$\begin{aligned} \begin{array}{l} x - k\left( \begin{array}{l} \frac{{\left( {1 - 2{\mu ^*}} \right) \left( {2{\mu ^*} + x} \right) }}{{r_1^3}} + \frac{{{\mu ^*}\left( {2{\mu ^*} + x - 1 + l } \right) }}{{r_{12}^3}} + {\mu ^*}\frac{{\mu \left( {2{\mu ^*} + x - 1 - l} \right) }}{{r_{22}^3}}\left( {1 + \frac{{3A}}{{2r_{22}^2}}} \right) \end{array} \right) =0,\vspace{0.3cm} \\ {1 - k\left( {\frac{{\left( {1 - 2\mu ^* } \right) }}{{r_1^3}} + \frac{\mu ^* }{{r_{21}^3}} + \frac{\mu ^* }{{r_{22}^3}}\left( {1 + \frac{{3A}}{{2r_{22}^2}}} \right) } \right) } =0. \end{array} \end{aligned}$$

In the absence of the disturbing forces, i.e. $l = 0$, and $A = 0$, the solution of Eq. (10) is given by $r_1=r_{12}=r_{22}=r_2= k^{2/3}$. Thus, the coordinates of the triangular equilibria can be obtained as $x=\frac{1}{2}\left( {1-4\mu ^*}\right)$ and $y=\pm \frac{1}{2}(4 k^{\frac{2}{3}}-1)$. If the perturbing forces influence the locations of these points, i.e. $l\ne 0$ and $A\ne 0$, the solutions of Eq. (10) are slightly changed by a very small quantity $\varepsilon$. Then the perturbed locations are given by $x = \frac{1}{2}\left( {1 - 4\mu ^* } \right) +{\varepsilon _1}$ and $y=\pm \frac{1}{2}(4 k^{\frac{2}{3}}-1)+{\varepsilon _2}$, $({\varepsilon _1}$, ${\varepsilon _2}$ $\ll 1)$. Substituting these values into Eq. (10), retaining only the first order terms in ${\varepsilon _1}$ and ${\varepsilon _2}$, and neglecting the higher orders, we have

11 $$\begin{aligned} \begin{array}{l} {A_1}({\mu ^*},k,A,l) + {A_2}({\mu ^*},k,A,l){\varepsilon _1} + {A_3}({\mu ^*},k,A,l){\varepsilon _2} = 0, \\ {B_1}({\mu ^*},k,A,l) + {B_2}({\mu ^*},k,A,l){\varepsilon _1} + {B_3}({\mu ^*},k,A,l){\varepsilon _2} = 0. \end{array} \end{aligned}$$

Solving simultaneously the two linear Eq. (11) give the values of $\varepsilon _1$ and $\varepsilon _2$. The quantities $A_1, A_2, A_3, B_1, B_2$, $B_3$, ${\varepsilon _1}$ and ${\varepsilon _2}$ are functions of the involved parameters and are given in Appendix I. The coordinates of the non-collinear equilibrium points $L_{4,5}(x, \pm y)$, are given as

12 $$\begin{aligned} \begin{array}{l} x = \frac{1}{2} - 2\mu ^*+({ -\frac{A}{4} -\frac{A^2}{16}}+ \left( {\frac{5}{{2{k^{2/3}}}} - \mu ^* - 2{\mu ^*{} ^2}} \right) \frac{{{A^2}}}{8} + \left( \begin{array}{l} \frac{5}{{2{k^{2/3}}}} - 1 - 2\mu ^* - 4{\mu ^*{} ^2} \end{array} \right) \frac{{Al}}{4}+ \cdots ),\\ \\ y = \pm \frac{{{{\left( {4{k^{2/3}} - 1} \right) }^{1/2}}}}{2}\mathrm{{ }} + {\sqrt{ - 1 + 4{k^{2/3}}} } \,(\, A + \left( \begin{array}{l} 1 - \frac{5}{{{k^{2/3}}}} - 2\mu ^* - 4{\mu ^*{} ^2} \end{array} \right) \frac{{{A^2}}}{4} + \left( \begin{array}{l} 1 - \frac{5}{{2{k^{2/3}}}} - 2\mu ^* - 4{\mu ^*{} ^2} \end{array} \right) Al+\cdots ). \end{array} \end{aligned}$$

It is clear from the above equations that the coordinates of the non-collinear equilibria are affected by the parameters *l*, $\mu ^*$, *A*, and *k*.

**Table 1:** The locations of the triangular points for some selected values of *l* and *k*, when $A = 0$, $\mu ^* = 0.149$.

A	l	k	*x*	$\pm y$
0	0	0.15	0.202	0.179752
0	0	0.5	0.202	0.616409
0	0	1	0.202	0.866025
0	0	1.5	0.201999	1.029743
0	0	1.85	0.202	1.121162
0	0.01	0.15	0.201899	0.180148
0	0.01	0.5	0.202022	0.616425
0	0.01	1	0.202058	0.866015
0	0.01	1.5	0.202073	1.029727
0	0.01	1.85	0.202079	1.121145
0	0.05	0.5	0.202561	0.616814
0	0.05	1	0.203474	0.865785
0	0.05	1.5	0.203842	1.029361
0	0.09	0.15	0.194784	0.206706
0	0.09	0.05	0.203897	0.617659
0	0.09	1	0.206823	0.865214
0	0.09	1.5	0.208000	1.0284789
0	0.09	1.85	0.208492	1.119776

**Table 2:** The locations of the triangular points for some selected values of *A*, *l*, *k*, and $\mu ^* = 0.149$.

A	l	k	*x*	$\pm y$
0.05	0	0.15	0.190655	0.208632
0.05	0	0.5	0.190068	0.625899
0.05	0	1	0.189869	0.872916
0.05	0	1.5	0.189785	1.035584
0.05	0	1.85	0.189749	1.126545
0.05	0.05	0.15	0.191711	0.207662
0.05	0.05	0.5	0.191983	0.624878
0.05	0.05	1	0.191942	0.872088
0.05	0.05	1.5	0.191903	1.034862
0.05	0.05	1.85	0.191884	1.125874
− 0.05	0	0.15	0.216273	0.133543
− 0.05	0	0.5	0.215185	0.605476
− 0.05	0	1	0.214915	0.858439
− 0.05	0	1.5	0.214812	1.023422
− 0.05	0	1.85	0.21477	1.115381
− 0.05	0.05	0.15	0.208357	0.165807
− 0.05	0.05	0.5	0.214049	0.607765
− 0.05	0.05	1	0.215714	0.858893
− 0.05	0.05	1.5	0.216366	1.023421
− 0.05	0.05	1.85	0.216636	1.115232

Tables 1, 2 represent the locations of the triangular equilibrium points for different combinations of the perturbing parameters. The selected values of the parameters are $\mu ^* = 0.149$, $A \in \{ -0.05, 0 , 0.05\}$, $l \in \{0, 0.01, 0.05, 0.09\}$, and $k \in \{0.15, 0.5, 1, 1.5, 1.85\}$. These parameters determine the potential distribution of the current dynamical system. We notice from the tables that the locations of the points vary with the values of the parameters. Changing the values of the parameters leads to a change in the gravitational field of the two massive primaries, consequently changing the coordinates of the points. The locations of the equilibrium points sometimes become close to one of the primaries, and sometimes move away from it according to the magnitude of the gravitational field. It is observed that, as *k* increases, *x* varies a little while *y* is displaced upward. Hence the libration point $L_4$ moves upward away from the x-axis in the xy-plane. For instance, in the Table 1, the shift (in the y-coordinate) between the location of point $L_4$ in the unperturbed case and its location when $A=0$, $k=1.5$, and $l=0.01$, is approximately 19 percent. When $k=0.5$, the variation in the y-coordinates is 28.8 percent, and the point $L_4$ shifted downward.

## Location of collinear libration points

In the case of collinear equilibrium points, $(L_i, i=1,2,3)$, $x\ne$0 and $y=0$. Therefore, ${\Omega _x}\left( {x,0} \right) = 0$, and the collinear equilibria lie on the line joining the two primary bodies, ($x-$axis). These points are the solution of the Eq. (9), so that

13 $$\begin{aligned} {\Omega _x}(x,0) = x - k\left( \begin{array}{l} \frac{{\left( {1 - 2{\mu ^*}} \right) \left( {x + 2{\mu ^*}} \right) }}{{{{\left| {x + 2{\mu ^*}} \right| }^3}}} + \frac{{\mu \left( {x + 2{\mu ^*} - 1 + l} \right) }}{{{{\left| {x + 2{\mu ^*} - 1 + l} \right| }^3}}} + \frac{{{\mu ^*}\left( {x + 2{\mu ^*} - 1 - l} \right) }}{{{{\left| {x + 2{\mu ^*} - 1 - l} \right| }^3}}}\left( {1 + \frac{{3A}}{{2{{\left| {x + 2{\mu ^*} - 1 - l} \right| }^2}}}} \right) \end{array} \right) =0 \end{aligned}$$

Equation (13) represents a nonlinear equation of *x* in a polynomial form. The highest degree of this nonlinear equation is eleven, and its solution is obtained using numerical methods. To guarantee the accuracy of the solution, we use Mathematica software to solve the equation with a high tolerance^31^. In the case of collinear point $L_1$, Eq. (13), reduces to

$$\begin{aligned} x + k\left( \begin{array}{l} \frac{{1 - 2{\mu ^*}}}{{{{\left( {x + 2{\mu ^*}} \right) }^2}}} + \frac{{{\mu ^*}}}{{{{\left( {x + 2{\mu ^*} + l - 1} \right) }^2}}} + \frac{{{\mu ^*}}}{{{{\left( {x + 2{\mu ^*} - 1 - l} \right) }^2}}} + \frac{{3{\mu ^*}A}}{{2{{\left( {x + 2{\mu ^*} - 1 - l} \right) }^4}}} \end{array} \right) ,x < - 2{\mu ^*}, \end{aligned}$$

while in the case of the points $L_2$ and $L_3$ Eq. (13) become, respectively,

$$\begin{aligned} x + k\left( \begin{array}{l} - \frac{{1 - 2{\mu ^*}}}{{{{\left( {x + 2{\mu ^*}} \right) }^2}}} + \frac{{{\mu ^*}}}{{{{\left( {x + 2{\mu ^*} + l - 1} \right) }^2}}} + \frac{{{\mu ^*}}}{{{{\left( {x + 2{\mu ^*} - 1 - l} \right) }^2}}} + \frac{{3{\mu ^*}A}}{{2{{\left( {x + 2{\mu ^*} - 1 - l} \right) }^4}}} \end{array} \right) , - 2{\mu ^*}< x < 1 - 2{\mu ^*} - l, \end{aligned}$$

and

$$\begin{aligned} x - k\left( \begin{array}{l} \frac{{1 - 2{\mu ^*}}}{{{{\left( {x + 2{\mu ^*}} \right) }^2}}} + \frac{{{\mu ^*}}}{{{{\left( {x + 2{\mu ^*} + l - 1} \right) }^2}}} + \frac{{{\mu ^*}}}{{{{\left( {x + 2{\mu ^*} - 1 - l} \right) }^2}}} + \frac{{3{\mu ^*}A}}{{2{{\left( {x + 2{\mu ^*} - 1 - l} \right) }^4}}} \end{array} \right) ,1 - 2{\mu ^*} - l < x. \end{aligned}$$

**Table 3:** Locations of the collinear points for different combinations of the system parameters when $A = 0$ and $\mu ^* = 0.149$.

l	k	$L_1$	$L_2$	$L_3$
0	0.15	− 0.69409	0.213996	0.929966
0	0.5	− 0.931225	0.272291	1.103415
0	1	− 1.12241	0.289075	1.257159
0	1.5	− 1.25826	0.295040	1.372302
0.01	1	− 1.12241	0.288887	1.257352
0.05	1	− 1.12246	0.284466	1.261948
0.09	1	− 1.12259	0.274656	1.272305
0.01	1.2	− 1.18108	0.291841	1.306526
0.05	1.2	− 1.18114	0.287272	1.310638
0.09	1.2	− 1.18127	0.277160	1.319966
0.05	1.5	− 1.25833	0.290116	1.376032
0.05	0.5	− 0.93126	0.268498	1.110579

**Table 4:** Locations of the collinear points for different combinations of the system parameters when $A = 0.05$ and $\mu ^*=0.149$.

l	k	$L_1$	$L_2$	$L_3$
0	0.5	− 0.931431	0.255729	1.133377
0	1	− 1.12269	0.269382	1.27884
0	1.2	− 1.18138	0.271759	1.326051
0	1.5	− 1.25859	0.274164	1.389722
0.01	1	− 1.12268	0.270733	1.280398
0.05	1	− 1.12271	0.271977	1.290481
0.09	1	− 1.12282	0.266612	1.306051
0.05	0.15	− 0.69420	0.206659	1.004532
0.05	0.35	− 0.85009	0.246656	1.097846
0.05	0.65	− 0.99832	0.264213	1.198195
0.05	0.9	− 1.09050	0.270348	1.265939
0.05	1.2	− 1.18141	0.274449	1.336239
0.05	1.5	− 1.25862	0.276952	1.398315
0.05	1.8	− 1.32641	0.278637	1.454374
0.05	2	− 1.36763	0.279485	1.489094

**Table 5:** Locations of the collinear points for different combinations of the system parameters when $A = -0.05$ and $\mu ^* = 0.149$.

l	k	$L_1$	$L_2$	$L_3$
0	0.5	− 0.93102	0.296075	1.048122
0	1	− 1.12213	0.320057	1.229039
0	1.2	− 1.18078	0.324525	1.281992
0	1.5	− 1.25794	0.3291507	1.351725
0.01	1	− 1.12213	0.315723	1.226615
0.05	1	− 1.12221	0.299931	1.218435
0.09	1	− 1.12236	0.283654	1.210615
0.05	0.15	− 0.69402	0.218330	0.674635
0.05	0.35	− 0.84979	0.266971	0.674698
0.05	0.65	− 0.99791	0.289644	0.674719
0.05	0.9	− 1.09001	0.297758	1.18590
0.05	1.2	− 1.18087	0.303236	1.275141
0.05	1.5	− 1.25803	0.306598	1.347598
0.05	1.8	− 1.32579	0.308871	1.410524
0.05	2	− 1.36699	0.310015	1.448712
0.09	0.65	−0.99802	0.275113	0.671187
0.01	0.65	−0.99784	0.303098	1.109130

Tables 3, 4, and 5 evaluate the effects due to the force ratio, the oblateness, and the distance parameters on the collinear equilibrium points. In these tables, we choose arbitrary values of the perturbing parameters *k* and *l*, with fixed values for $\mu ^*$ and *A*.

In Table 3 when $k = 0.5$, $A=0$, $l=0$, and $\mu ^*=0.149$ the point $L_1$ approaches the bigger primary, and the change in its location is 17 percent. Also, the point $L_2$ tends toward the origin and approaches the bigger primary, and the change in its location is 5 percent. The point $L_3$ approaches the smaller one, and the change in its location is 12 percent. However, the situation reverses in the case of $k =1.5$. In this case, the variations in the points $L_1, L_2$, and $L_3$ are 12, 2, and 9 percent, respectively. The variations are measured relative to the case with $k=1$, $A=0$, $l=0$, and $\mu ^*=0.149$. When *l* $\in \{0.01, 0.05, 0.09 \}$ with fixed $k=1.2$, $A=0$, and $\mu ^*=0.149$, we notice that the values of the points $L_1$, $L_2$, and $L_3$ are changed. This demonstrates the importance of the influence of the perturbing parameters on the locations of the points. Similarly, we can describe Tables 4 and 5 in the two cases $A=\pm 0.05$. The significance of the oblateness coefficient is clear from the values of the collinear points in the two tables.

**Table 6:** Locations of the collinear points for different mass parameter when $A =\pm 0.05$, $k = 1$ and $l = 0.05$.

*A*	$\mu ^*$	$L_1$	$L_2$	$L_3$
0.05	0.05	− 1.04170	0.587097	1.308373
0.05	0.1	− 1.08303	0.419027	1.309973
0.05	0.15	− 1.12351	0.269067	1.289964
0.05	0.2	− 1.16248	0.126146	1.260064
0.05	0.24	− 1.19195	0.013889	1.232025
− 0.05	0.05	− 1.04155	0.622145	0.872732
− 0.05	0.1	− 1.08271	0.450024	1.114779
− 0.05	0.15	− 1.12301	0.296964	0.992380
− 0.05	0.2	− 1.16176	0.151490	0.885905
− 0.05	0.24	− 1.19103	0.037414	0.803177

**Table 7:** Locations of the collinear points for different mass parameter when $A =\pm 0.05$, $k=1.5$ and $l=0.05$.

*A*	$\mu ^*$	$L_1$	$L_2$	$L_3$
0.05	0.05	− 1.18392	0.598776	1.392177
0.05	0.1	− 1.22231	0.426924	1.408065
0.05	0.15	− 1.25934	0.273986	1.397971
0.05	0.2	− 1.29434	0.128420	1.375820
0.05	0.24	− 1.32017	0.014139	1.352974
− 0.05	0.05	− 1.18374	0.637763	1.204076
− 0.05	0.1	− 1.22193	0.460521	1.084806
− 0.05	0.15	− 1.25875	0.303561	0.980949
− 0.05	0.2	− 1.29348	0.154761	0.879245
− 0.05	0.24	− 1.31908	0.038214	0.79843

**Table 8:** Locations of the collinear points for different mass parameter when $A =\pm 0.05$, $k=0.5$ and $l=0.05$.

*A*	$\mu ^*$	$L_1$	$L_2$	$L_3$
0.05	0.05	− 0.83953	0.551021	1.212205
0.05	0.1	− 0.88591	0.396026	1.188618
0.05	0.15	− 0.93237	0.255083	1.150711
0.05	0.2	− 0.97838	0.119754	1.106693
0.05	0.24	− 1.01433	0.013189	1.069166
− 0.05	0.05	− 0.83942	0.574858	0.872669
− 0.05	0.1	− 0.88567	0.420097	0.772701
− 0.05	0.15	− 0.93201	0.278543	0.672712
− 0.05	0.2	− 0.97787	0.142432	0.572717
− 0.05	0.24	− 1.01371	0.035205	0.492720

Tables 6, 7, and 8 are devoted to showing the effect of the mass ratio $\mu ^*$ on the points. For arbitrarily chosen values of the mass parameters $\mu ^*$, the locations of the collinear points are presented in Tables 6, 7 and 8 with different values of the parameters *k*. In Table 6, we compute the locations of the collinear points when $k = 1$, $l = 0.05$, and $A = \pm 0.05$. When $A=0.05$ and increasing $\mu ^*$ from 0.05 to 0.24, the point $L_1$ moves away from the bigger primary, and the change in its location is approximately 12 percent. The point $L_2$ moves toward the bigger primary, and its location changes by approximately 97 percent. The point $L_3$ approaches the smaller primary, and the variation of its location is 5.8 percent. In the case of $A=-0.05$, the variations will not differ significantly. These variations are close to the results obtained by Idrisi et al.^25^. Similarly, we can discuss Tables 7 and 8 for the two values $k = (1.5, 0.5)$.

## Stability of motion around equilibrium points

Examining the stability of the equilibrium points is essential for dynamical systems because they represent suitable positions for constructing periodic orbits. Consequently, gives valuable insights into the dynamics of celestial objects. Not all equilibrium points are stable due to the influence of various disturbance forces. So, it is necessary to study the stability of these points^32^.

To investigate the linear stability of the equilibria, it is necessary to transfer the origin of the coordinate system to the position of the obtained libration points. After that, we linearize the equations of the dynamical system around these equilibrium points. To do that, let us denote the coordinates of the equilibrium point to be $(x_{0}, y_{0})$, then we give the point a small displacement (*X*, *Y*), we have

14 $$\begin{aligned} \begin{array}{l} X = {x_0} - x\vspace{.05cm}\\ Y = {y_0} - y \end{array} \end{aligned}$$

Substituting Eq. (14) into Eq. (1) and using Taylor series, we get

15 $$\begin{aligned} \begin{array}{l} {\ddot{X}} - 2\omega \dot{Y} = \Omega _x^0 + \frac{1}{{1!}}\left( {X \frac{\partial }{{\partial x}} + Y \frac{\partial }{{\partial y}}} \right) \Omega _x^0 + \frac{1}{{2!}}{\left( {X \frac{\partial }{{\partial x}} + Y \frac{\partial }{{\partial y}}} \right) ^2}\Omega _x^0 + O(3)\\ {\ddot{Y}} - 2\omega \dot{X} = \Omega _y^0 + \frac{1}{{1!}}\left( {X \frac{\partial }{{\partial x}} + Y \frac{\partial }{{\partial y}}} \right) \Omega _y^0 + \frac{1}{{2!}}{\left( {X \frac{\partial }{{\partial x}} + Y \frac{\partial }{{\partial y}}} \right) ^2}\Omega _y^0 + O(3) \end{array} \end{aligned}$$

retaining only terms up to order one of *X* and *Y*, then the linear variational equations of motion become

16 $$\begin{aligned} \begin{array}{l} {\ddot{X}} - 2\omega \dot{Y} = X \Omega _{xx}^0 + \Omega _{xy}^0 Y\vspace{.05cm}\\ {\ddot{Y}} + 2\omega \dot{X} = X \Omega _{xy}^0 + \Omega _{yy}^0 Y \end{array} \end{aligned}$$

where $\Omega _{xx}^0$, $\Omega _{yy}^0$, and $\Omega _{xy}^0$ represent the partial derivatives evaluated at one of the equilibrium points $(x_0, y_0)$. The characteristic equation corresponding to Eq. (16) can be written in the form

17 $$\begin{aligned} {\lambda ^4} + \left( {4{\omega ^2} - \Omega _{xx}^0 - \Omega _{yy}^0} \right) {\lambda ^2} + \Omega _{xx}^0\Omega _{yy}^0 - {\left( {\Omega _{xy}^0} \right) ^2} = 0 \end{aligned}$$

where

$$\begin{aligned} \Omega _{xx}^0= & {} {\omega ^2}\left( {1 - k\left( \begin{array}{l} \frac{{\left( {1 - 2{\mu ^*}} \right) }}{{r_1^{*3}}}\left( {1 - \frac{{3{{\left( {{x_0} + 2{\mu ^*}} \right) }^2}}}{{r_1^{*2}}}} \right) + \frac{{{\mu ^*}}}{{r_{12}^{*3}}}\left( {1 - \frac{{3{{\left( {{x_0} + 2{\mu ^*} - 1 + l} \right) }^2}}}{{r_{12}^{*2}}}} \right) \\ + \frac{{{\mu ^*}}}{{r_{22}^{*3}}}\left( \begin{array}{l} 1 - \frac{{3\left( { - A + 2{{({x_0} + 2{\mu ^*} - 1 - l)}^2}} \right) }}{{2r_{22}^{*2}}} - \frac{{15A{{\left( {{x_0} + 2{\mu ^*} - 1 - l} \right) }^2}}}{{2r_{22}^{*4}}} \end{array} \right) \end{array} \right) } \right) \\ \Omega _{yy}^0= & {} {\omega ^2}\left( {1 - k\left( \begin{array}{l} \frac{{\left( {1 -2{\mu ^*}} \right) }}{{r_1^{*3}}}\left( {1 - \frac{{3{y^2}}}{{r_1^{*2}}}} \right) + \frac{{{\mu ^*}}}{{r_{12}^{*3}}}\left( {1 - \frac{{3{y^2}}}{{r_{12}^{*2}}}} \right) + \frac{{{\mu ^*}}}{{r_{22}^{*3}}}\left( {1 + \frac{{3\left( {A - 2{y^2}} \right) }}{{2r_{22}^{*2}}} -\frac{{15A{y^2}}}{{2r_{22}^{*4}}}} \right) \end{array} \right) } \right) \end{aligned}$$

and

$$\begin{aligned} \Omega _{xy}^0 = 3k{\omega ^2}{y_0}\left( \begin{array}{l} \frac{{\left( {1 - 2{\mu ^*}} \right) \left( {{x_0} + 2{\mu ^*}} \right) }}{{r_1^{*5}}} + \frac{{{\mu ^*}\left( {{x_0} + 2{\mu ^*} - 1 + l} \right) }}{{r_{12}^{*5}}} + \frac{{{\mu ^*}\left( {{x_0} + 2{\mu ^*} - 1 - l} \right) }}{{r_{22}^{*5}}}\left( {1 + \frac{{5A}}{{2r_{22}^{*2}}}} \right) \end{array} \right) \end{aligned}$$

where

$$\begin{aligned} \begin{array}{l} r _{1}^* = \sqrt{{{\left( {{x_0} + 2{\mu ^*}} \right) }^2} + y_0^2} \vspace{0.3cm}\\ r _{21}^* = \sqrt{{{\left( {{x_0} + 2{\mu ^*} - 1 + l} \right) }^2} + y_0^2} \vspace{0.3cm}\\ r _{22}^* = \sqrt{{{\left( {{x_0} + 2{\mu ^*} - 1 - l} \right) }^2} + y_0^2} \end{array} \end{aligned}$$

A libration point will be linearly stable if Eq. (17), evaluated at the point, has complex roots with negative real parts or four purely imaginary roots. The libration point is classified as unstable if one or more of the eigenvalues have a positive real part^33–35^.

**Table 9:** Numerical locations of some stable collinear points with $\mu ^* = 0.149$, $A = -0.05$, $l\in \{0,0.01,0.05,0.09\}$, and $k \in \{0.5, 1, 1.5\}$.

$L_{2}$	$\lambda _{1,2}$	$\lambda _{3,4}$	$L_{3}$	$\lambda _{1,2}$	$\lambda _{3,4}$	Stability
0.499805	± 7.489406 i	± 1.701609 i	0.901205	± 8.010691 i	± 1.135279 i	Stable
0.496685	± 8.584259 i	± 2.438210 i	0.921496	± 4.176862 i	± 1.274633 i	Stable
0.524428	± 8.858407 i	± 2.365751 i	0.896976	± 10.49689 i	± 0.623891 i	Stable
0.534155	± 10.67172 i	± 1.262657 i	1.062499	± 3.748642 i	± 0.694272 i	Stable
0.671754	± 17.97117 i	± 13.65656 i	0.922021	± 7.132201 i	± 0.559539 i	Stable
0.671257	± 18.53528 i	± 11.19270 i	0.994578	± 5.025379 i	± 0.360998 i	Stable
0.498288	± 7.946184 i	± 2.027790 i	0.899003	± 9.088204 i	± 0.972129 i	Stable
0.671874	± 14.49949 i	± 11.31217 i	0.999777	± 4.468936 i	± 0.626283 i	Stable
0.671377	± 14.89128 i	± 9.488392 i	0.934239	± 4.816854 i	± 1.067109 i	Stable
0.671187	± 15.06449 i	± 8.646110 i				Stable
0.527727	± 7.674159 i	± 1.596446 i				Stable
0.531779	± 8.281013 i	± 1.213610 i				Stable

## Stability of collinear points

For the collinear libration points $y = 0$, so ${r_1} = \left| {x + 2{\mu ^*}} \right|$, ${r_{21}} = \left| {x + 2{\mu ^*} - 1 + l} \right|$, and ${r_{22}} = \left| {x + 2{\mu ^*} - 1 - l} \right|$. Also, the necessary and sufficient conditions for these points to be stable are that the following conditions are satisfied simultaneously

$$\left( 4{\omega ^2} - \Omega _{xx}^0 - \Omega _{yy}^0)^2 \right) -4\Omega _{yy}^0 \Omega _{xx}^0 >0$$,    $$4 n^2 - \Omega _{xx}^0-\Omega _{yy}^0 > 0$$,    $$\Omega _{xx}^0 \Omega _{yy}^0>0$$.

If any one of the mentioned conditions is not satisfied, this will necessarily lead to unbounded motion around the points. Since $\Omega _{xy}^0 =\Omega _{yx}^0= 0$, so that the characteristic equation of the linearized system for the collinear points reduces to

18 $$\begin{aligned} {\lambda ^4} + \left( {4{\omega ^2} - \Omega _{xx}^0 - \Omega _{yy}^0} \right) {\lambda ^2} + \Omega _{xx}^0\Omega _{yy}^0 = 0 \end{aligned}$$

where

19 $$\begin{aligned} \begin{array}{l} \Omega _{xx}^0 = {\omega ^2}\left( {1 + 2k\left( {\frac{{\left( {1 - 2{\mu ^*}} \right) }}{{r_1^3}} + \frac{{{\mu ^*}}}{{r_{12}^3}} + \frac{{{\mu ^*}}}{{r_{22}^3}} + \frac{{3{\mu ^*}A}}{{r_{22}^5}}} \right) } \right) \\ \Omega _{yy}^0 = {\omega ^2}\left( {1 - k\left( {\frac{{\left( {1 - 2{\mu ^*}} \right) }}{{r_1^3}} + \frac{{{\mu ^*}}}{{r_{12}^3}} + \frac{{{\mu ^*}}}{{r_{22}^3}} + \frac{{3{\mu ^*}A}}{{2r_{22}^2}}} \right) } \right) \end{array}\ \end{aligned}$$

Let $\Delta = {\lambda ^2}$, so Eq. (18) can be written in the form

20 $$\begin{aligned} {\Delta ^2} + b\Delta + c = 0 \end{aligned}$$

then the solutions of Eq. (20), can be written as

21 $$\begin{aligned} {\Delta _{1,2}} = - \frac{1}{2}\left[ {b \pm \sqrt{{b^2} - 4c} } \right] \end{aligned}$$

where

$$\begin{aligned} \begin{array}{l} \lambda _{1,2}= \pm \sqrt{\Delta _1} \hspace{0.5cm} \lambda _{3,4}= \pm \sqrt{\Delta _2} \end{array} \end{aligned}$$

and

22 $$\begin{aligned} \begin{array}{l} b={4{\omega ^2} - \Omega _{xx}^0 - \Omega _{yy}^0}\vspace{.05cm}\\ c=\Omega _{xx}^0\Omega _{yy}^0 \end{array} \end{aligned}$$

**Figure 3:**
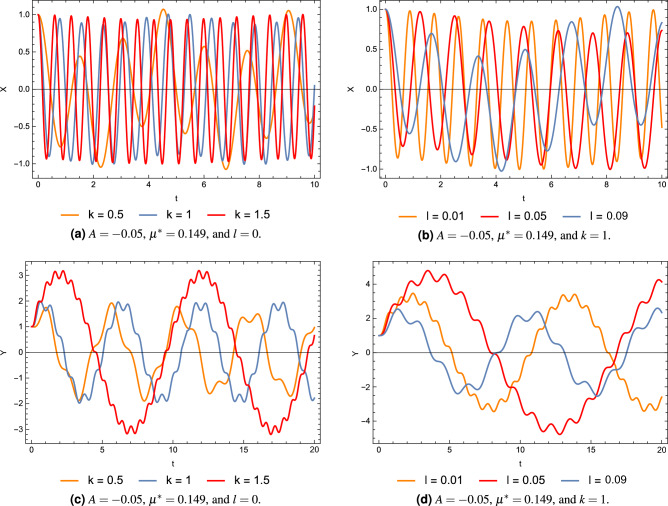
Solutions for the perturbed motion in case of different perturbations.

**Figure 4:**
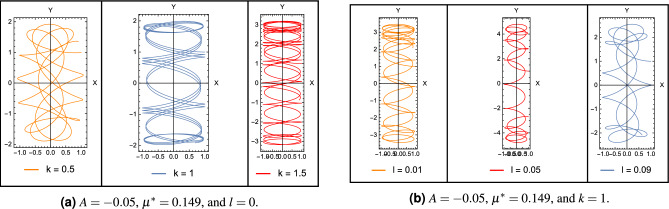
Trajectories around stable collinear points for $A=-0.05$, and different combinations of the system parameters.

The general solution of the system of linear differential Eq. (16) with constant coefficients can be written as^33^

23 $$\begin{aligned} \begin{array}{l} X(t)=\alpha _1 e^{\lambda _1 t}+\alpha _2 e^{\lambda _2 t}+\alpha _3 e^{\lambda _3 t}+\alpha _4 e^{\lambda _4 t} \\ Y(t)=\beta _1 e^{\lambda _1 t}+\beta _2 e^{\lambda _2 t}+\beta _3 e^{\lambda _3 t}+\beta _4 e^{\lambda _4 t} \end{array} \end{aligned}$$

where the constants $\alpha _i$ $(i = 1,2,3,4)$, are functions of the four arbitrary constants $\beta _i$ $(i = 1,2,3,4)$. The relationship between $\alpha _i$ and $\beta _i$ can be derived from Eq. (16). For more details see Appendix II.

As mentioned above, the nature of the roots given by the characteristic equation, $\lambda _{1,2}$ and $\lambda _{3,4}$ determine the dynamic behavior of the system. In other words, determine whether the collinear points are stable or not. Table 9 contains some numerical locations of the collinear points $L_{2,3}$ in case of different combinations of the involved system parameters. The locations are calculated for the values $k \in \{0.5, 1, 1.5\}$, $l \in \{0, 0.01, 0.05, 0.09\}$ with fixed $\mu ^*=0.149$, and $A=-0.05$.

In the case of the positive oblateness parameter, the values of the roots of the characteristic equation are not purely imaginary and thus the stability conditions are not satisfied. Similarly, when $A=0$ the conditions are not satisfied^23,36^. When $A = - 0.05$, some stable locations for the collinear points are obtained. The characteristic equation has four imaginary eigenvalues $\lambda _{1,2}=\pm i \sqrt{\Delta _1}$ and $\lambda _{3,4}=\pm i\sqrt{\Delta _2}$, where $\Delta _1$ and $\Delta _2$ are real numbers. Numerical calculations are performed for different combinations of the system parameters to investigate the stability of the points, see Table 9.

Figure 3 illustrates the solution for the perturbed motion *X*(*t*) and *Y*(*t*) for the obtained stable locations. We observe from the figures that the solutions are regular without any exponential growth, but we can see a shift between the solutions and different amplitudes. This is due to the difference in the perturbing parameters taken into account. Figure 4 illustrates the trajectories around the collinear point $L_3$ when $A=-0.05$. The figures show how changing system parameters affect the shape of the trajectories. These trajectories correspond to the stable collinear equilibrium points that we obtained.

## Stability of non-collinear points

To examine the stability of the equilateral libration points, let us consider the libration point ${L_4}\left( {{x_0},{y_0}} \right)$. For this point we have

$$\begin{aligned} \begin{array}{l} x_0 = \frac{1}{2} - 2\mu ^*+ {\varepsilon _1},\\ y_0 = \pm \frac{{{{\left( {4{k^{2/3}} - 1} \right) }^{1/2}}}}{2}\mathrm{{ }} + {\varepsilon _2}, \end{array} \end{aligned}$$

**Figure 5:**
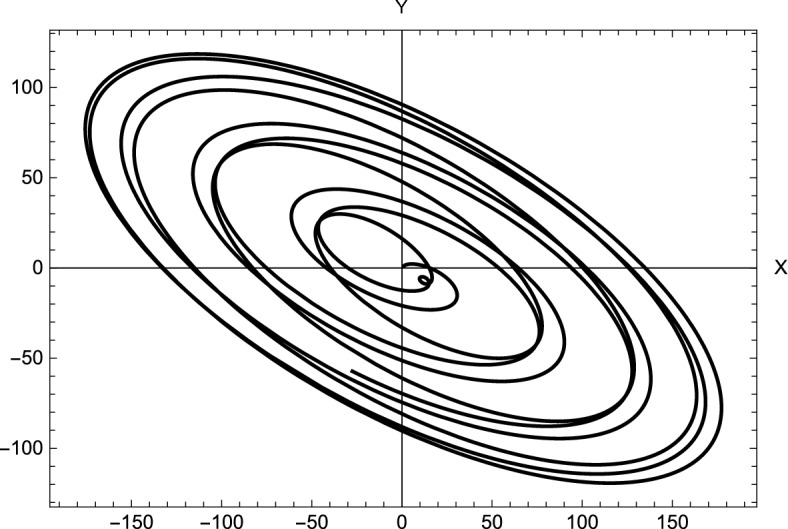
The unperturbed solution around the equilibrium point $L_4$ ( $A = 0$, $k = 1$, $l = 0$ and $\mu ^*=0.019 )$.

**Figure 6:**
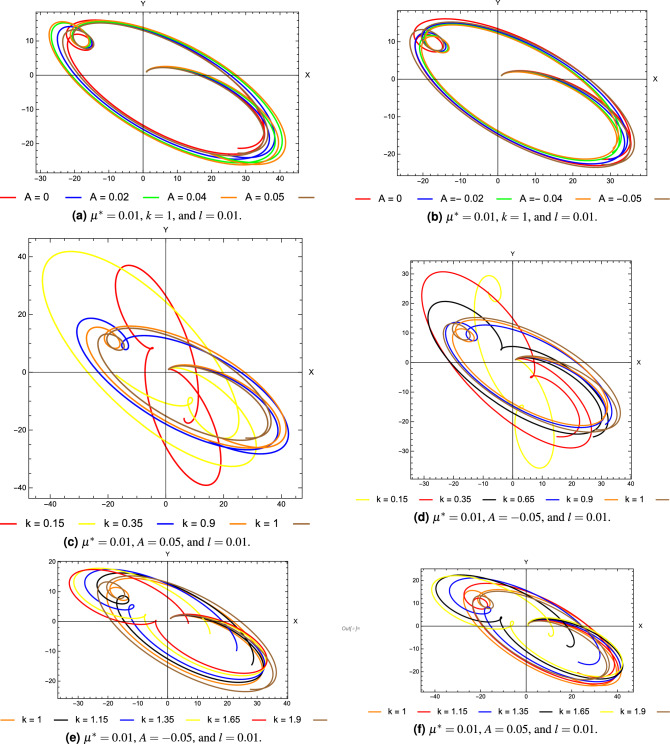
The perturbed solutions around the point $L_4$ for different combinations of the system parameters.

Substituting ${\lambda ^2} = \Lambda$, then the characteristic Eq. (17) at the non-collinear point can be written as

24 $$\begin{aligned} {\Lambda ^2} + b\Lambda + c = 0 \end{aligned}$$

Equation (24) represents a second-degree algebraic equation with coefficients *b* and *c*. Where

$$\begin{aligned} \begin{array}{l} b = 4{\omega ^2} - \Omega _{xx}^0 - \Omega _{yy}^0,\vspace{.07cm} \\ c = \Omega _{xx}^0\Omega _{yy}^0 - {(\Omega _{xy}^0)^2},\vspace{.07cm} \\ Q=b-4 c^2. \end{array} \end{aligned}$$

and *Q* represents the discriminant. The quantities $\Omega _{xx}^0$, $\Omega _{yy}^0$, $\Omega _{xy}^0$, *b*, *c*, and *Q* are given in Appendix III. The roots of Eq. (24) are given as

25 $$\begin{aligned} {\Lambda _{1,2}} = \frac{1}{2}\left[ {-b \pm \sqrt{Q} } \right] \end{aligned}$$

where $\lambda _{1,2}=\pm \sqrt{{\Lambda _1}} \,\,\,\,\,$, $\lambda _{3,4}=\pm \sqrt{\Lambda _2}$. In the interval $0<\mu ^*<\frac{1}{2}$, if $Q=b^2-4c>0$ then (24) gives four distinct pure imaginary roots which lead to a stable motion around the triangular points. When $Q<0$, the real parts of two of the roots are positive, thus the triangular points are unstable. Also if $Q=0$, we have double roots and this leads to secular terms in the solutions, and the motion around the triangular points is unstable.

Now the discriminant *Q* can be written as a function of the mass parameter $\mu ^*$ in the form

26 $$\begin{aligned} Q = \alpha {\mu ^{*2}} + \beta {\mu ^*} + \gamma \end{aligned}$$

where the quantities $\alpha$, $\beta$, and $\gamma$ are given in Appendix III. When $k=1$, then

**Figure 7:**
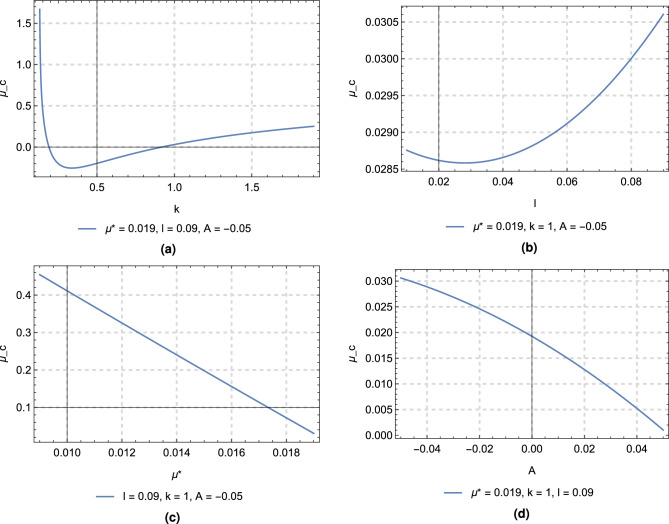
Variation of the critical mass ratio with the perturbing parameters.

27 $$\begin{aligned} {Q}= & {} (1 - 54\mu ^* + 108{\mu ^*{}^2}) + (3 - 186\mu ^* + 360{\mu ^*{} ^2})A + (1 - \frac{{2337\mu ^* }}{{16}} + \frac{{2193{\mu ^*{} ^2}}}{8}){A^2} + (42\mu ^* - 153{\mu ^*{}^2})Al \nonumber \\{} & {} +(\frac{{15}}{4} - \frac{{619\mu ^* }}{{16}} - \frac{{379{\mu ^*{} ^2}}}{8}){A^2}l + (\frac{{15}}{4} + \frac{{1133\mu ^* }}{{16}} + \frac{{587{\mu ^*{} ^2}}}{8})A{l^2} \nonumber \\{} & {} + ( - \frac{{25}}{8} + \frac{{13033\mu ^* }}{{32}} - \frac{{26773{\mu ^*{} ^2}}}{{32}}){A^2}{l^2} + (\frac{{81\mu ^* }}{2} + 9{\mu ^*{} ^2}){l^2}. \end{aligned}$$

For instance, in the case $A=0$, $l=0$, and $Q=0$, the mass ratio $\mu _c^*= \mu ^*= 0.0192604$. For $A \ne 0$, and $l\ne 0$ the critical mass given as

$$\begin{aligned} \mu _c^*\mathrm{{ = 0}}\mathrm{{.0192604}} - 0.448888A - 1.71153{A^2} + 0.752179Al + 2.98729{A^2}l + 0.783385{l^2} + 5.1411A{l^2} + 4.40903{A^2}{l^2}. \end{aligned}$$

Figure 5 represents the perturbed solution around the triangular point $L_4$ with null system parameters and $\mu ^*=0.019$. Figure 6 depict some selected perturbed solutions around the point $L_4$ for different perturbing parameters and $\mu ^*=0.01$. The figures show slightly different sizes and shapes due to differences in the system parameters. In the case $\mu ^*\le \mu _c^{*}$, the solutions show a bounded and periodic nature, while otherwise the motion around the points $L_{4,5}$ is unstable.

Putting $Q=0$ in Eq. (26) and solving, we obtain the critical mass ratio of the current dynamical system, $\mu _c^{*}$. Its value depends on the perturbing parameters, the mass ratio $\mu ^{*}$, the oblateness coefficient *A*, the force ratio *k*, and the distance *l*. Figure 7a–d show the variation of the critical mass ratio against the parameters $k \in [0.13, 1.9]$, $l \in [0.01, 0.09]$, $A\in [-0.05, 0.05]$, and $\mu ^* \in [0.009, 0.019]$, respectively. The figures show that all the perturbing parameters play a significant role in determining the value of $\mu _c^{*}$.

## Application to the 2001 SN263 asteroid system

Asteroids are remnant objects from the beginning of the solar system. Shapes and sizes of asteroids vary significantly, ranging from small rocks to dwarf planets. Most of these celestial objects are located in the region between the orbits of Mars and Jupiter in the main asteroid belt. The 2001 SN263 asteroid system is one of the famous triple systems in the near-Earth population^37,38^. Exploring the dynamical characteristics in the environment around the components of this system, such as studying the zero velocity curves and orbits around the libration points, is crucial for interplanetary space missions. So, we use the 2001 SN263 asteroid system as an application for the current mathematical model.

We first define the dynamical model to begin studying the dynamics. We assume that the more massive body $M_1$ (Alpha) has a spherical shape, while the second asteroid $M_2$ (Gamma) has an irregular shape and is modeled as a rotating mass dipole. The rotation period of the body $M_2$ around its axis is equal to the orbital period of the asteroids around their barycenter. In this case, we have a restricted synchronous four-body problem^36^. Numerical simulations are conducted assuming that the mass parameter $\mu ^*=0.005284$ and the mass dipole has a fixed size with $l=0.0657203$.

**Figure 8:**
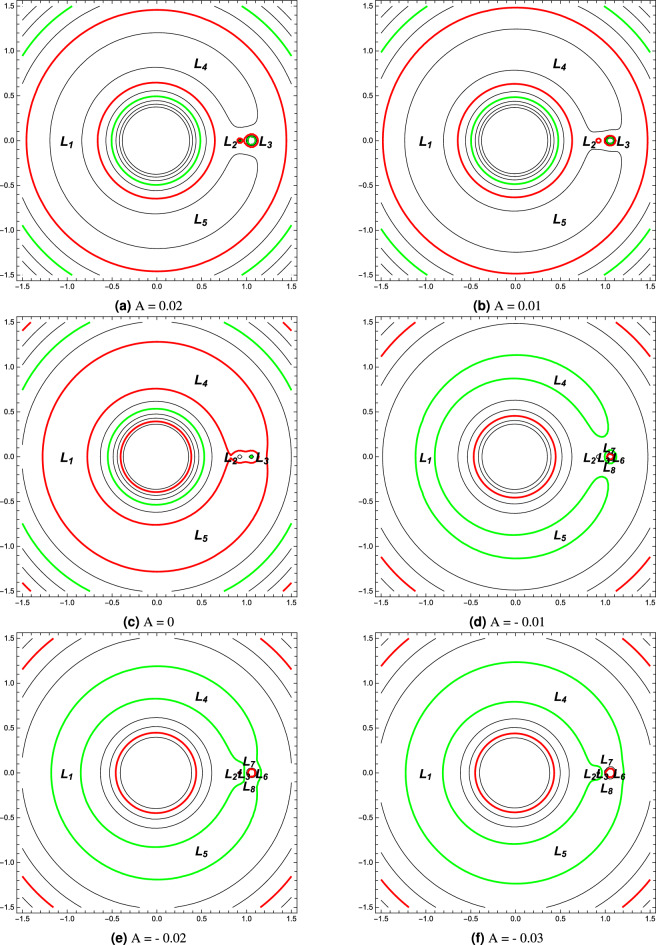
Curves of zero velocity for different oblateness parameter.

The regions of motion around the equilibrium points are plotted for different values of the oblateness parameter *A*, with the fixed values $\mu =0.005284$, $k=1$, and $l=0.06572$. The location of the equilibrium points and associated zero-velocity curves for different values of the oblateness parameter are given in Fig. 8. At $A=0.02$, we observe that a circular region around the massive primary $M_1$ appears where the motion of the small object is possible (Fig. 8a). However, The small object can’t reach the libration points. Decreasing the value of the oblateness parameter $A=0.01$, the situation remains the same except for a relative increase in the size of the forbidden zone around the equilibrium points $L_1, L_2$, and $L_3$ (Fig. 8b). When $A=0$, the picture is slightly different. A contact occurs between the two regions around the two primaries. However, the third body can’t move to infinity (Fig. 8c). We also note that in the cases $A=0, \quad A = 0.01$, and $A=0.02$, the number of the equilibrium points is similar to that of the classical restricted three-body problem. In the case of negative values of the oblateness parameter, the shape of the contours did not differ significantly from the case of its positive values, but the difference is clear in the number of equilibrium points that accumulated around the small primary (Fig. 8d–f).

**Figure 9:**
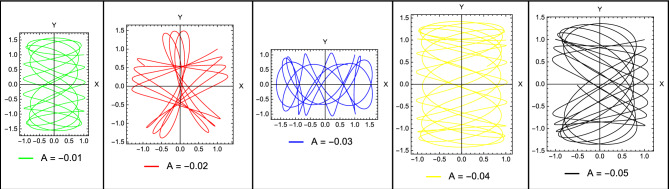
Trajectories around $L_3$ collinear point in the stable cases for the asteroid system.

**Table 10:** Locations of collinear and triangular points of the 2001 SN263 asteroid system. $\mu =0.005284$, $k=1$, and $l=0.06572$.

A	$L_1$(x,0)	$L_2$(x,0)	$L_3$(x,0)	$L _{4,5}$ ( x, $\pm y$)
− 0.05	− 1.004398	0.831197	0.959645	(0.503043, $\pm$ 0.858202)
− 0.04	− 1.004400	0.829226	0.962242	(0.500565, $\pm$ 0.859631)
− 0.03	− 1.004401	0.827305	0.965754	(0.49813, $\pm$ 0.8610355)
− 0.02	− 1.004403	0.825432	0.971017	(0.495736, $\pm$ 0.862416)
− 0.01	− 1.004404	0.823608	0.980855	(0.493385,$\pm$ 0.863772)
0.00	− 1.004406	0.821832	1.172872	(0.491075, $\pm$ 0.865104)
0.01	− 1.004407	0.820102	1.197908	(0.488807, $\pm$ 0.866411)
0.02	− 1.004409	0.818417	1.211191	(0.486581, $\pm$ 0.867694)
0.03	− 1.0044105	0.816775	1.220799	(0.484397, $\pm$ 0.868953)
0.04	− 1.0044119	0.815176	1.228483	(0.482255, $\pm$ 0.870188)
0.05	− 1.0044134	0.813618	1.234955	(0.480154, $\pm$ 0.871398)

**Figure 10:**
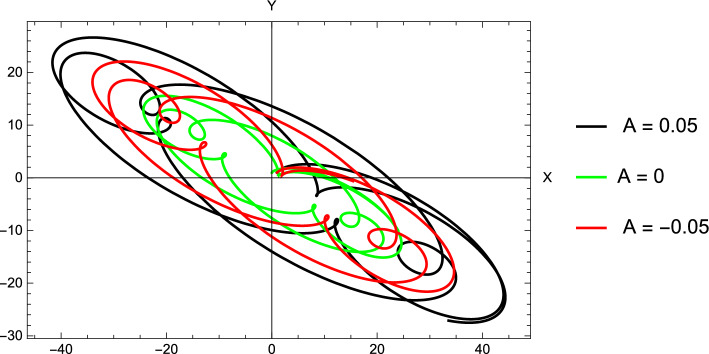
Trajectories around the non-collinear point $L_4$ for the asteroids system.

The numerical locations of $L_i, (i=1, 2, 3, 4, 5)$ of the 2001 SN263 asteroid system are presented in Table 10, for different values of the parameter *A*. The table shows that the effect of the oblateness parameter is significant in the two points $L_2$ and $L_3$, while its effect in $L_1$ is less significant. This is because $L_2$ and $L_3$ are close to the dipole which has an irregular shape. The collinear points are all unstable in the case of the positive oblateness parameter and the case $A=0$. In the case of negative values, we found some stable points $L_3$. These stable cases are plotted in Fig. 9. Table 10, also shows the locations of the triangular points $L_{4,5}$. The locations are calculated for different values of the parameter *A*. We can observe the variation in the locations with changing *A*. It may be noted that for $A=0$, our results for the locations of collinear and triangular points of the asteroid 2001 SN263 are in complete agreement with Santos et al.^13^ and Idrisi et al.^25^. If we consider the oblateness parameter, this will lead to a change in the locations of the collinear point $L_3$. For instance, if $A=-0.05$, the point approaches the smaller primary, and the variation of its location is 18.7 percent. Also, if $A=0.05$, the point moves away from the smaller primary, and the variation of its location is 5.3 percent. Where the calculations are performed in comparison to the case $A=0$. In the triangular points case, when $A=-0.05$, the *x* and *y* coordinates of the point $L_4$ vary little. The point moves downward towards the x-axis in the xy-plane, and the change of its y-coordinates is approximately 0.8 percent. In the case $A=0.05$, the point moves upward and the change of its y-coordinates is approximately 0.7 percent. For all values of *A*, the four roots of the characteristic equation are purely imaginary. Thus, the non-collinear libration points for the current asteroid system are linearly stable. These results entirely agree with Santos et al.^13^.

Figure 9, depicts the Lissajous trajectories associated with the collinear equilibrium points. The trajectories are plotted for different negative values of the oblateness parameter. The figure shows that, as the oblateness parameter changes the shape and the size of each trajectory change. Also, it affects the time the orbit takes to complete one period. Figure 10 depicts the motion around the point $L_4$ for different values of the parameter *A*. The figure shows the looping nature of the trajectory of the third body in the rotating frame. It is clear from the figure that although the different orbits are similar in shape, the perturbing parameter *A* changes the size of the orbits and their orbital period.

## Summary and conclusions

Studying the dynamics around small objects represents a major challenge for those working in the field of space science. This is due to the difficulty of modeling the gravitational field of these objects and the lack of information available about the true shape of these objects. Therefore, deepening the study to understand the dynamics and investigate stability around these objects is essential for future spaceflights. The current work is a contribution to understand and clarify the behavior of spaceships around these small objects. The investigations are conducted within the restricted four-body problem frame of work.

To achieve the mentioned goals, we formulated the dynamical equations of motion of the small body under the perturbations considered. The current force model comprises the influence of the gravitational field of the two primary bodies. The massive primary is assumed to have a spherical shape, while the small primary is modeled as a rotating mass dipole. The parameters that determine the gravitational field of the mass dipole are the oblateness parameter, the force ratio, and the distance parameter. The present model is a generalization of the traditional dipole shape. To clarify the dynamics, different initial conditions are used for the system parameters to carry out several numerical simulations.

One of the important studies in this research is the study of surfaces of zero velocity. These surfaces provide us with valuable and important information about the regions of motion between the main bodies and the equilibrium points. Also, it determines the possibility of transfer of the small body between the equilibrium points and the main bodies. We found that the system parameters affect the structure of the curves of zero velocity. In the case of the negative oblateness parameter, new equilibrium points exist near the mass dipole. Also, it is seen that the possibility of transfer depends on the combinations of the perturbing parameters.

In addition, we investigated the existence and stability of the equilibrium points. We have found that the locations of the collinear points may approach or move away from the main bodies according to the values of the system parameters. Similarly, the locations of the triangular points may approach or move away from the x-axis according to the values of the parameters. This is because any change in the parameters inevitably leads to a change in the gravitational field of the primary bodies. We also found that the points $L_2$ and $L_3$ are more affected than the point $L_1$. In the case of the negative oblateness parameter, new equilibrium points exist near the mass dipole. Regarding the stability of the collinear points, we found some stable points correspond to the negative values of the oblateness parameter. Otherwise, these points are unstable. Furthermore, the triangular points $L_{4,5}$ are stable in the range $0<\mu ^*\le \mu _c^*$, $\mu _c^*=0.019$, regardless of the values of the remaining parameters.

As an application to the current theory, we studied the effect of the perturbing forces taken into account on the location and stability of the equilibrium points of the 2001 SN263 asteroid system, as well as their effect on the curves of zero velocity. The results show that the effect of the oblateness parameter is significant in the collinear points $L_2$ and $L_3$, while its effect in $L_1$ is less significant. This is a result of the proximity of these two points to the mass dipole. Also, the locations of the triangular points change with each change in the values of the oblateness parameter. Furthermore, we found that the collinear points are all unstable in the case of the positive oblateness parameter and the case A = 0. However, we found some stable points for negative values in the case of $L_3$. The non-collinear points for the asteroid system are linearly stable.

## Supplementary Information

Supplementary Information.

## Data Availability

All data generated or analysed during this study are included in this published article.
