# Supplementary material for: Dynamics around small irregularly shaped objects modeled as a mass dipole
Source: Sci Rep. 2024 May 23;14:11764. doi: 10.1038/s41598-024-61821-w (PMC11688459; doi:10.1038/s41598-024-61821-w)
Supplement: Supplementary file 1 — Supplementary Information. [file 41598_2024_61821_MOESM1_ESM.pdf]

Appendix(I)

The quantities in Eqs. (11) can be written as

$$A_1 = \frac{3A\mu^*}{4k^{2/3}} + \frac{3A\left(-5+4k^{2/3}\right)l\mu^*}{8k^{4/3}} - \frac{\left(-35+60k^{2/3}\right)3Al^2\mu^*}{32k^2} - \frac{\left(-5+12k^{2/3}\right)3l^2\mu^*}{8k^{4/3}},$$

$$A_2 = \frac{3}{4k^{2/3}} - \frac{3A\left(-5+4k^{2/3}\right)\mu^*}{8k^{4/3}} + \frac{15A\left(-7+12k^{2/3}\right)l\mu^*}{16k^2} + \frac{\left(35-120k^{2/3}+48k^{4/3}\right)3l^2\mu^*}{16k^2} + \frac{\left(315-840k^{2/3}+240k^{4/3}\right)3Al^2\mu^*}{64k^{8/3}},$$

$$A_3 = b_0\ \left(\frac{3}{4k^{2/3}} + \left(-\frac{15A}{8k^{4/3}} - \frac{3}{k^{2/3}}\right)\mu^* - \frac{15A\left(-7+4k^{2/3}\right)l\mu^*}{16k^2} + \frac{\left(-63+84k^{2/3}\right)15Al^2\mu^*}{64k^{8/3}} + \frac{\left(-7+12k^{2/3}\right)15l^2\mu^*}{16k^2}\right),$$

$$B_1 = -\frac{3A\mu^*}{2k^{2/3}} + \frac{15Al\mu^*}{4k^{4/3}} + \frac{\left(-35+20k^{2/3}\right)3Al^2\mu^*}{16k^2} + \frac{\left(-5+4k^{2/3}\right)3l^2\mu^*}{4k^{4/3}},$$

$$B_2 = \frac{3}{2k^{2/3}} + \left(-\frac{15A}{4k^{4/3}} - \frac{6}{k^{2/3}}\right)\mu^* - \frac{15A\left(-7+4k^{2/3}\right)l\mu^*}{8k^2} + \frac{\left(-63+84k^{2/3}\right)15Al^2\mu^*}{32k^{8/3}} + \frac{\left(-7+12k^{2/3}\right)15l^2\mu^*}{8k^2},$$

$$B_3 = b_0\left(\frac{3}{2k^{2/3}} + \frac{15A\mu^*}{4k^{4/3}} - \frac{105Al\mu^*}{8k^2} - \frac{15\left(-63+28k^{2/3}\right)Al^2\mu^*}{32k^{8/3}} - \frac{15\left(-7+4k^{2/3}\right)l^2\mu^*}{8k^2}\right).$$

$$\varepsilon_1 = \left(\begin{array}{l}
-\frac{A}{4} - \frac{A^2}{16} + \left(\frac{5}{2k^{2/3}} - \mu^* - 2\mu^{*2}\right)\frac{A^2}{8} + \left(\frac{5}{2k^{2/3}} - 1 - 2\mu^* - 4\mu^{*2}\right)\frac{Al}{4} \\
+\left(\frac{5}{k^{2/3}} - \frac{15}{2k^{4/3}} - \frac{1}{4} - \mu^* + \frac{5\mu^*}{k^{2/3}} - 3\mu^{*2} + \frac{10\mu^{*2}}{k^{2/3}}\right)\frac{A^2l}{4} + \left(1 - \frac{5}{8k^{2/3}} + \mu^* + 2\mu^{*2}\right)l^2 \\
+\left(1 - \frac{135}{32k^{2/3}} + \frac{365}{32k^2} - \frac{55}{16k^{4/3}} + \frac{17\mu^*}{4} - \frac{175\mu^*}{16k^{4/3}} - \frac{15\mu^*}{8k^{2/3}} + \frac{27\mu^{*2}}{2} - \frac{175\mu^{*2}}{8k^{4/3}} - \frac{25\mu^{*2}}{8k^{2/3}}\right)\frac{A^2l^2}{4} \\
+\left(1 + \frac{5}{4k^{4/3}} - \frac{7}{2k^{2/3}} - \frac{\mu^*}{k^{2/3}} + \frac{12\mu^*}{5} + \frac{28\mu^{*2}}{5} - \frac{2\mu^{*2}}{k^{2/3}}\right)\frac{5Al^2}{8}
\end{array}\right),$$

and

$$\varepsilon_2 = b_0\left(\begin{array}{l}
A + \left(1 - \frac{5}{k^{2/3}} - 2\mu^* - 4\mu^{*2}\right)\frac{A^2}{4} + \left(1 - \frac{5}{2k^{2/3}} - 2\mu^* - 4\mu^{*2}\right)Al \\
+\left(\frac{1}{4} + \frac{15}{2k^{4/3}} - \frac{5}{k^{2/3}} - \mu^{*2} + \frac{5\mu^*}{k^{2/3}} + \frac{10\mu^{*2}}{k^{2/3}}\right)A^2l + \left(\frac{5}{2k^{2/3}} - 4 + 8\mu^{*2} + 4\mu^*\right)l^2 \\
+\left(\frac{35}{4k^{2/3}} - \frac{5}{2} - \frac{25}{8k^{4/3}} + 4\mu^* - \frac{5\mu^*}{2k^{2/3}} + 10\mu^{*2} - \frac{5\mu^{*2}}{k^{2/3}}\right)Al^2 \\
+\left(-1 - \frac{365}{32k^2} + \frac{55}{16k^{4/3}} + \frac{135}{32k^{2/3}} - \frac{\mu^*}{4} - \frac{175\mu^*}{16k^{4/3}} - \frac{35\mu^{*2}}{8k^{2/3}} - \frac{5\mu^*}{2k^{2/3}} + \frac{7\mu^{*2}}{2} - \frac{175\mu^{*2}}{8k^{4/3}}\right)A^2l^2
\end{array}\right).$$

where

$$b_0 = \sqrt{-1+4k^{2/3}}.$$

Appendix(II)

Eqs. (16) can be written in matrix form as

$$\begin{pmatrix} \dot{X} \\ \dot{Y} \\ \ddot{X} \\ \ddot{Y} \end{pmatrix} = \begin{pmatrix} 0 & 0 & 1 & 0 \\ 0 & 0 & 0 & 1 \\ \Omega^0_{xx} & \Omega^0_{xy} & 0 & 2 \\ \Omega^0_{xy} & \Omega^0_{yy} & -2 & 0 \end{pmatrix} \begin{pmatrix} X \\ Y \\ \dot{X} \\ \dot{Y} \end{pmatrix}$$

which can be written as

$$\mathbf{X} = \mathbf{A}\dot{\mathbf{X}}$$

where

$$\mathbf{X} = \begin{pmatrix} X \\ Y \\ \dot{X} \\ \dot{Y} \end{pmatrix}, \qquad \mathbf{A} = \begin{pmatrix} 0 & 0 & 1 & 0 \\ 0 & 0 & 0 & 1 \\ \Omega^0_{xx} & \Omega^0_{xy} & 0 & 2 \\ \Omega^0_{xy} & \Omega^0_{yy} & -2 & 0 \end{pmatrix}$$

The above equations are coupled and it is much better to use a suitable transformation to a new set of n variables, Y_i, $(i = 1, 2, \ldots, n)$. Where $\dot{Y}_i$ is a function of Y_i alone. Let the transformation from $\mathbf{X}$ to $\mathbf{Y}$ be represented by

$$\mathbf{Y} = \mathbf{B}\mathbf{X}$$

where $\mathbf{B}$ is a constant matrix. Now we have $\mathbf{X} = \mathbf{B}^{-1}\mathbf{Y}$ and $\dot{\mathbf{X}} = \mathbf{B}^{-1}\dot{\mathbf{Y}}$. Then we can write the solutions for X, Y, $\dot{X}$ and $\dot{Y}$ as

$$X = \sum_{i=1}^{4} \alpha_i e^{\lambda_i t} \qquad \dot{X} = \sum_{i=1}^{4} \alpha_i \lambda_i e^{\lambda_i t}$$

$$Y = \sum_{i=1}^{4} \beta_i e^{\lambda_i t} \qquad \dot{Y} = \sum_{i=1}^{4} \beta_i \lambda_i e^{\lambda_i t}$$

Substituting X,Y,$\dot{X}$,and $\dot{Y}$ into Eqs. (16)

$$\sum_{i=1}^{4} \left(\alpha_i \lambda_i^2 - 2\beta_i \lambda_i - \Omega_{xx}^0 \alpha_i - \Omega_{xy}^0 \beta_i\right) e^{\lambda_i t} = 0$$

from which

$$\beta_i = \frac{\lambda_i^2 - \Omega_{xx}^0}{2\lambda_i + \Omega_{xy}^0} \alpha_i$$

If, at $t = 0$, we have the initial conditions $X = X_0$, $Y = Y_0$, $\dot{X} = \dot{X}_0$ and $\dot{Y} = \dot{Y}_0$. Then the quantities α_i and β_i are obtained from the solution of the four simultaneous linear equations

$$\sum_{i=1}^{4} \alpha_i = X_0 \qquad \sum_{i=1}^{4} \beta_i = Y_0$$

$$\sum_{i=1}^{4} \alpha_i \lambda_i = \dot{X}_0 \qquad \sum_{i=1}^{4} \beta_i \lambda_i = \dot{Y}_0$$

then we can derive the solution

$$X(t) = \alpha_1 e^{\lambda_1 t} + \alpha_2 e^{\lambda_2 t} + \alpha_3 e^{\lambda_3 t} + \alpha_4 e^{\lambda_4 t}$$

$$Y(t) = \beta_1 e^{\lambda_1 t} + \beta_2 e^{\lambda_2 t} + \beta_3 e^{\lambda_3 t} + \beta_4 e^{\lambda_4 t}$$

Appendix(III)

The partial derivatives Ω_{xx}^0, Ω_{yy}^0, Ω_{xy}^0, b, c, and Q can be written as

$$\Omega_{xx}^0 = \frac{3}{4}\left(\begin{array}{c} \frac{1}{k^{2/3}} + \frac{A}{2k^{2/3}} + \frac{5Al}{2k^{4/3}} - \frac{Al}{k^{2/3}} - \frac{5l^2}{2k^{4/3}} + \frac{4l^2}{k^{2/3}} + \frac{15A^2\mu^*}{16k^2} - \frac{25A^2\mu^*}{4k^{4/3}} + \frac{13A^2\mu^*}{2k^{2/3}} + \\ \frac{4A\mu^*}{k^{2/3}} - \frac{5A\mu^* l}{2k^2} + \frac{2A\mu^* l}{k^{2/3}} + \frac{5l^2\mu^*}{2k^2} - \frac{10l^2\mu^*}{k^{4/3}} - \frac{4l^2\mu^*}{k^{2/3}} + \frac{A^2\mu^{*2}}{k^{2/3}} + \frac{4Al\mu^{*2}}{k^{2/3}} - \frac{8l^2\mu^{*2}}{k^{2/3}} \end{array}\right)$$

$$+A^2 a_1\left(-30 + 141k^{2/3} - 132k^{4/3}\right) + 2A^2 l a_2(180 - 825k^{2/3} + 660k^{4/3} - 96k^2)$$

$$+2Al^2 a_2(-75 + 495k^{2/3} - 1056k^{4/3} + 528k^2) + 4A^2 l\mu^{*2} a_1(75 - 336k^{2/3} + 240k^{4/3})$$

$$+A^2 l^2 a_3(-1095 + 4785k^{2/3} - 930k^{4/3} - 1824k^2 - 768k^{8/3}) + 2A^2 l\mu^* a_3(-45 - 510k^{2/3}$$

$$+3660k^{4/3} - 3720k^2 + 864k^{8/3}) + 2Al^2\mu^* a_3(-375 + 2580k^{2/3} - 5280k^{4/3} + 4272k^2 - 2496k^{8/3})$$

$$+A^2 l^2 \mu^* a_4(2085 - 12945k^{2/3} + 31290k^{4/3} - 70500k^2 + 71664k^{8/3} - 11904k^{10/3})$$

$$+16Al^2\mu^{*2} a_1(-15 + 90k^{2/3} - 168k^{4/3}) + A^2 l^2 \mu^{*2} a_2(-255 - 840k^{2/3} + 9384k^{4/3} - 7584k^2),$$

$$\Omega_{yy}^{0}=3\left(\begin{array}{l}1-\frac{1}{4k^{2/3}}+\frac{3A}{2}-\frac{A}{8k^{2/3}}-\frac{5Al}{8k^{4/3}}+\frac{Al}{4k^{2/3}}+\frac{5l^2}{8k^{4/3}}-\frac{l^2}{k^{2/3}}-\frac{15A^2\mu^*}{64k^2}+\frac{5A^2\mu^*}{8k^{4/3}}-\frac{A^2\mu^*}{8k^{2/3}}\\+\frac{5Al\mu^*}{8k^2}-\frac{5Al\mu^*}{2k^{4/3}}-\frac{Al\mu^*}{2k^{2/3}}-\frac{5l^2\mu^*}{8k^2}+\frac{5l^2\mu^*}{2k^{4/3}}+\frac{l^2\mu^*}{k^{2/3}}-\frac{A^2\mu^{*2}}{4k^{2/3}}-\frac{Al\mu^{*2}}{k^{2/3}}+\frac{2l^2\mu^{*2}}{k^{2/3}}\end{array}\right)$$

$$+A^2a_1(30-141k^{2/3}+120k^{4/3})+2A^2la_2(-180+825k^{2/3}-600k^{4/3}+72k^2)$$
$$+2Al^2a_2(75-495k^{2/3}+996k^{4/3}-432k^2)+A^2l^2a_3(1095-4785k^{2/3}+1230k^{4/3}+324k^2+1632k^{8/3})$$
$$+A^2l\mu^*a_3(45-630k^{2/3}+1980k^{4/3}-552k^2-960k^{8/3})+2Al^2\mu^*a_3(375-3120k^{2/3}+7440k^{4/3}$$
$$-4368k^2+2688k^{8/3})+A^2l^2\mu^*a_4(-2085+20265k^{2/3}-62670k^{4/3}+76380k^2-68736k^{8/3}+14400k^{10/3})$$
$$+4A^2l\mu^{*2}a_1(-75+348k^{2/3}-264k^{4/3})+16A^2l\mu^{*2}a_1(15-96k^{2/3}+180k^{4/3})+A^2l^2\mu^{*2}a_2(255+900k^{2/3}$$
$$-10848k^{4/3}+8928k^2),$$

and

$$\Omega_{xy}^{0}=\frac{3b_0}{4k^{2/3}}(1-4\mu^*)+\frac{3A}{b_0}(1-\frac{1}{8k^{2/3}})+\frac{3A^2}{8b_0}(-7-\frac{5}{2k^{4/3}}+\frac{27}{4k^{2/3}})+\frac{3Al}{2b_0}(-1-\frac{5}{4k^{4/3}}+\frac{3}{k^{2/3}})$$
$$+\frac{A^2l}{8b_0}(-21+\frac{45}{k^2}-\frac{465}{4k^{4/3}}+\frac{105}{k^{2/3}})+\frac{3l^2}{b_0}(2+\frac{5}{8k^{4/3}}-\frac{9}{4k^{2/3}})+\frac{Al^2}{4b_0}(51-\frac{75}{8k^2}+\frac{345}{8k^{4/3}}-\frac{159}{2k^{2/3}})$$
$$+\frac{A^2l^2}{a_3b_0}(1095-6975k^{2/3}+10650k^{4/3}+1014k^2-11448k^{8/3}+3648k^{10/3})+\frac{3A\mu^*}{b_0}(-6+\frac{1}{k^{2/3}})$$
$$+\frac{3A^2\mu^*}{b_0}(-\frac{1}{4}+\frac{1}{k^{2/3}}-\frac{5}{8k^{4/3}}+\frac{15}{64k^2})+\frac{3Al\mu^*}{b_0}(-1-\frac{1}{2k^{2/3}}-\frac{5}{8k^2}+\frac{15}{4k^{4/3}})+\frac{A^2l\mu^*}{b_0}(-6+\frac{45}{64k^{8/3}}$$
$$-\frac{405}{32k^2}+\frac{15}{8k^{4/3}}+\frac{87}{8k^{2/3}})+\frac{3l^2\mu^*}{b_0}(2+\frac{2}{k^{2/3}}-\frac{15}{4k^{4/3}}+\frac{5}{8k^2})+\frac{Al^2\mu^*}{b_0}(18+\frac{375}{64k^{8/3}}-\frac{225}{8k^2}+\frac{225}{8k^{4/3}}-\frac{51}{2k^{2/3}})$$
$$+\frac{A^2l^2\mu^*}{a_4b_0}(2085-24285k^{2/3}+89100k^{4/3}-135420k^2+158880k^{8/3}-79968k^{10/3}+20352k^4)$$
$$+\frac{3A^2\mu^{*2}}{2b_0}(-1+\frac{1}{2k^{2/3}})+\frac{9A^2l\mu^{*2}}{2b_0}(-3+\frac{7}{k^{2/3}}-\frac{5}{2k^{4/3}})+\frac{3Al\mu^{*2}}{b_0}(\frac{1}{k^{2/3}}-2)+\frac{Al^2\mu^{*2}}{b_0}(39+\frac{45}{4k^{4/3}}-\frac{93}{2k^{2/3}})$$
$$+\frac{6l^2\mu^{*2}}{b_0}(-\frac{1}{k^{2/3}}+2)+\frac{A^2l^2\mu^{*2}}{2a_2b_0}(-165-150k^{2/3}+6834k^{4/3}-15312k^2+6624k^{8/3}).$$

$$b=1+\frac{3A}{2}+(-\frac{A}{k^{2/3}}+\frac{15A^2}{16k^{4/3}}-\frac{3A^2}{2k^{2/3}}+\frac{5Al}{2k^{4/3}})3\mu^*+3A^2b_1+3A^2lb_2(-5+2k^{2/3})+3Al^2b_2(5-8k^{2/3})$$
$$+A^2l^2a_1(-75+375k^{2/3}-216k^{4/3})+4A^2l\mu^*a_2(285-1410k^{2/3}+1068k^{4/3}+24k^2)$$
$$+4Al^2\mu^*a_2(135-540k^{2/3}+24k^{4/3}-48k^2)+2A^2l^2\mu^*a_3(-1830+7845k^{2/3}-1470k^{4/3}-732k^2$$
$$-624k^{8/3})+12A^2l\mu^{*2}b_2(-1+2k^{2/3})+24Al^2\mu^{*2}b_2(1-2k^{2/3})+6A^2l^2\mu^{*2}a_1(-5+112k^{2/3}-112k^{4/3}),$$

$$c=\frac{9\mu^*}{k^{4/3}}(-\frac{1}{2}-A)+\frac{9\mu^*}{k^{2/3}}(2+6A)+3Al\mu^*(\frac{15}{8k^{8/3}}-\frac{105}{8k^2}+\frac{9}{k^{4/3}})+3l^2\mu^*(-\frac{15}{8k^{8/3}}+\frac{15}{k^2}-\frac{45}{2k^{4/3}}+\frac{6}{k^{2/3}})+\frac{9\mu^{*2}}{k^{4/3}}(1+2A)$$
$$+\frac{9\mu^{*2}}{k^{2/3}}(-4-12A)+3Al\mu^{*2}(-\frac{15}{4k^{8/3}}+\frac{45}{2k^2}-\frac{6}{k^{4/3}})+9l^2\mu^{*2}(\frac{5}{4k^{8/3}}-\frac{15}{2k^2}+\frac{6}{k^{4/3}})+9A^2b_1+9A^2lb_2(-5+2k^{2/3})$$
$$+9Al^2b_2(5-8k^{2/3})+A^2l^2a_1(-225+1395k^{2/3}-1080k^{4/3})+2A^2\mu^*a_3(135-810k^{2/3}+1764k^{4/3}-5040k^2$$
$$+10368k^{8/3})+4A^2l\mu^*a_4(135-4050k^{2/3}+19890k^{4/3}-28512k^2+15120k^{8/3}+288k^{10/3})$$
$$+4Al^2\mu^*a_4(1125-9270k^{2/3}+19440k^{4/3}+7776k^2-36576k^{8/3}+13248k^{10/3})+A^2l^2\mu^*c_1(-6255$$
$$+79830k^{2/3}-328815k^{4/3}+556200k^2-513864k^{8/3}+105840k^{10/3}+30528k^4)+2A^2\mu^{*2}a_3(-270$$
$$+1800k^{2/3}-4464k^{4/3}+10944k^2-20736k^{8/3})+A^2l\mu^{*2}a_4(-270+6300k^{2/3}-27900k^{4/3}$$
$$+33264k^2-11808k^{8/3}+576k^{10/3})+Al^2\mu^{*2}a_4(-2250+19440k^{2/3}-46440k^{4/3}+14688k^2+12096k^{8/3}$$
$$-1152k^{10/3})+A^2l^2\mu^{*2}c_1(12510-149310k^{2/3}+598590k^{4/3}-1044720k^2+1026576k^{8/3}-239328k^{10/3}$$
$$-23040k^4),$$

and

$$Q=1+3A+\frac{9\mu^*}{k^{4/3}}(2+4A)+(-\frac{72}{k^{2/3}}-\frac{222A}{k^{2/3}})\mu^*+Al\mu^*(-\frac{45}{2k^{8/3}}+\frac{315}{2k^2}-\frac{93}{k^{4/3}})+9\mu^{*2}(-\frac{4}{k^{4/3}}-\frac{8A}{k^{4/3}})$$
$$+9l^2\mu^*(\frac{5}{2k^{8/3}}-\frac{20}{k^2}+\frac{30}{k^{4/3}}-\frac{8}{k^{2/3}})+12\mu^{*2}(\frac{12}{k^{2/3}}+\frac{36A}{k^{2/3}})+3l^2\mu^{*2}(-\frac{15}{k^{8/3}}+\frac{90}{k^2}-\frac{72}{k^{4/3}})$$
$$+9Al\mu^{*2}(\frac{5}{k^{8/3}}-\frac{30}{k^2}+\frac{8}{k^{4/3}})+24A^2b_1(-2+3k^{2/3})+30A^2lb_2(5-2k^{2/3})+30Al^2b_2(-5+8k^{2/3})$$
$$+2A^2l^2a_1(375-2325k^{2/3}+1800k^{4/3})+8A^2\mu^*a_3(-135+810k^{2/3}-1854k^{4/3}+5688k^2-11520k^{8/3})$$
$$+16A^2l\mu^*a_4(-135+4050k^{2/3}-19320k^{4/3}+25332k^2-11544k^{8/3}-240k^{10/3})$$
$$+16Al^2\mu^*a_4(-1125+9270k^{2/3}-19170k^{4/3}-8856k^2+36624k^{8/3}-13344k^{10/3})$$
$$+4A^2l^2\mu^*c_1(6255-79830k^{2/3}+325155k^{4/3}-538890k^2+503724k^{8/3}-105864k^{10/3}-32352k^4)$$
$$+8A^2\mu^{*2}a_3(270-1800k^{2/3}+4320k^{4/3}-10368k^2+20736k^{8/3})+16A^2l\mu^{*2}a_4(270-6300k^{2/3}$$
$$+28620k^{4/3}-36144k^2+11760k^{8/3}--480k^{10/3})+16Al^2\mu^{*2}a_4(2250-19440k^{2/3}+46440k^{4/3}-$$
$$-14688k^2-12000k^{8/3}+960k^{10/3})+4A^2l^2\mu^{*2}c_1(-12510+149310k^{2/3}-605430k^{4/3}+1072080k^2$$
$$-1027272k^{8/3}+243984k^{10/3}+19200k^4).$$

with

$$a_1=\frac{1}{-32k^{4/3}+128k^2}\qquad a_2=\frac{1}{-64k^2+256k^{8/3}}$$
$$a_3=\frac{1}{-128k^{8/3}+512k^{10/3}}\qquad a_4=\frac{1}{-256k^{10/3}+1024k^4}$$
$$b_0=\sqrt{-1+4k^{2/3}}\qquad b_1=\frac{1}{-8+32k^{2/3}}$$
$$b_2=\frac{1}{-8k^{2/3}+32k^{4/3}}\qquad c_1=\frac{1}{-256k^4+1024k^{14/3}}$$

Also, the quantities α, β, and γ can be written as

$$\alpha=(-\frac{36}{k^{4/3}}+\frac{144}{k^{2/3}})+A(-\frac{72}{k^{4/3}}+\frac{432}{k^{2/3}})+8A^2a_3(270-1800k^{2/3}+4320k^{4/3}-10368k^2+20736k^{8/3})+Al(\frac{45}{k^{8/3}}-\frac{270}{k^2}+\frac{72}{k^{4/3}})$$
$$+16A^2la_4(270-6300k^{2/3}+28620k^{4/3}-36144k^2+11760k^{8/3}-480k^{10/3})+l^2(-\frac{45}{k^{8/3}}+\frac{270}{k^2}-\frac{216}{k^{4/3}})$$
$$+16Al^2a_4(2250-19440k^{2/3}+46440k^{4/3}-14688k^2-12000k^{8/3}+960k^{10/3})$$
$$+4A^2l^2c_1(-12510+149310k^{2/3}-605430k^{4/3}+1072080k^2-1027272k^{8/3}+243984k^{10/3}+19200k^4),$$

$$\beta = (\tfrac{18}{k^{4/3}} - \tfrac{72}{k^{2/3}}) + (\tfrac{36}{k^{4/3}} - \tfrac{222}{k^{2/3}})A + 4A^2 a_3(-135 + 810k^{2/3} - 1854k^{4/3} + 5688k^2 - 11520k^{8/3})$$
$$+Al(-\tfrac{45}{2k^{8/3}} + \tfrac{315}{2k^2} - \tfrac{93}{k^{4/3}}) + 16A^2 l a_4(-135 + 4050k^{2/3} - 19320k^{4/3} + 25332k^2 - 11544k^{8/3} - 240k^{10/3})$$
$$+l^2(\tfrac{45}{2k^{8/3}} - \tfrac{180}{k^2} + \tfrac{270}{k^{4/3}} - \tfrac{72}{k^{2/3}}) + 16Al^2 a_4(-1125 + 9270k^{2/3} - 19170k^{4/3} - 8856k^2 + 36624k^{8/3} - 13344k^{10/3})$$
$$+4A^2 l^2 c_1(6255 - 79830k^{2/3} + 325155k^{4/3} - 538890k^2 + 503724k^{8/3} - 105864k^{10/3} - 32352k^4),$$

and

$$\gamma = 1 + 3A + 24A^2 b_1(-2 + 3k^{2/3}) + 30A^2 l b_2(5 - 2k^{2/3}) + 30Al^2 b_2(-5 + 8k^{2/3}) + 2A^2 l^2 a_1(375 - 2325k^{2/3} + 1800k^{4/3}).$$
